# Exploring Secondary Metabolite Profiles of *Stachybotrys* spp. by LC-MS/MS

**DOI:** 10.3390/toxins11030133

**Published:** 2019-02-27

**Authors:** Annika Jagels, Viktoria Lindemann, Sebastian Ulrich, Christoph Gottschalk, Benedikt Cramer, Florian Hübner, Manfred Gareis, Hans-Ulrich Humpf

**Affiliations:** 1 Institute of Food Chemistry, Westfälische Wilhelms-Universität Münster, 48149 Münster, Germany; a_jage01@uni-muenster.de (A.J.); v_lind05@uni-muenster.de (V.L.); cramerb@uni-muenster.de (B.C.); fhueb_01@uni-muenster.de (F.H.); 2 Chair of Food Safety, Veterinary Faculty, Ludwig-Maximilians-Universität München, 85764 Oberschleißheim, Germany; ulrich@ls.vetmed.uni-muenchen.de (S.U.); Christoph.Gottschalk@ls.vetmed.uni-muenchen.de (C.G.); m.gareis@ls.vetmed.uni-muenchen.de (M.G.)

**Keywords:** *Stachybotrys* spp., metabolite profiles, LC-MS/MS, satratoxins, phenylspirodrimanes, stachybotrychromenes, biosynthetic production

## Abstract

The genus *Stachybotrys* produces a broad diversity of secondary metabolites, including macrocyclic trichothecenes, atranones, and phenylspirodrimanes. Although the class of the phenylspirodrimanes is the major one and consists of a multitude of metabolites bearing various structural modifications, few investigations have been carried out. Thus, the presented study deals with the quantitative determination of several secondary metabolites produced by distinct *Stachybotrys* species for comparison of their metabolite profiles. For that purpose, 15 of the primarily produced secondary metabolites were isolated from fungal cultures and structurally characterized in order to be used as analytical standards for the development of an LC-MS/MS multimethod. The developed method was applied to the analysis of micro-scale extracts from 5 different *Stachybotrys* strains, which were cultured on different media. In that process, spontaneous dialdehyde/lactone isomerization was observed for some of the isolated secondary metabolites, and novel stachybotrychromenes were quantitatively investigated for the first time. The metabolite profiles of *Stachybotrys* species are considerably influenced by time of growth and substrate availability, as well as the individual biosynthetic potential of the respective species. Regarding the reported adverse effects associated with *Stachybotrys* growth in building environments, combinatory effects of the investigated secondary metabolites should be addressed and the role of the phenylspirodrimanes re-evaluated in future research.

## 1. Introduction

The genus *Stachybotrys* belongs to the family of Stachybotryaceae, phylum ascomycota and comprises a diversity of species known to possess saprotrophic potential [1]. This filamentous fungus can be found in nature as well as in the indoor environment, and cellulose-rich substrates containing high moisture levels are generally required for growth [2,3]. Fungal growth in water-damaged buildings poses serious human health risks and can lead to allergic and respiratory issues [4]. Regarding the latter, *Stachybotrys chartarum* (previous designations S. *atra* or *S. alternans*) has been the subject of considerable attention due to transitory associations with idiopathic pulmonary hemorrhage in infants [5,6] and disease symptoms called sick building syndrome [7,8]. There are still controversial discussions concerning the role of *Stachybotrys* within these reported cases, concluding that there is inadequate evidence that exposure to mycotoxins is causally related to the adverse human health effects [9,10]. Nevertheless, *Stachybotrys* produces a broad variety of toxic secondary metabolites, which are of relevance in the indoor environment concerning human health [11,12]; moreover, these toxins caused stachybotryotoxicosis in animals due to contaminated feed [13,14,15] and culinary herbs were also found to be contaminated by *Stachybotrys* sp. [16]. The *Stachybotrys* mycotoxins can be divided into 3 structural groups, macrocyclic trichothecenes (MCTs, e.g. satratoxins), atranones, and phenylspirodrimanes (PSDs) [17]. The most common species among this genus are *S. chlorohalonata* and *S. chartarum*, which were also found in the indoor environment. For the latter, two distinct chemotypes (CT) are known: *S. chartarum* CT S is capable of producing MCTs, such as satratoxins, roridins, and verrucarins, whereas the CT A biosynthesizes atranones and their precursors, the dolabellanes [18]. Remarkably, the PSDs are synthesized by both CTs, and not only by *S. chlorohalonata*, but generally by nearly all species and in notably higher levels than other secondary metabolites [17]. Due to the fact that MCTs are known to be severely toxic (LD_50_ in mice < 1 mg/kg), to inhibit the protein synthesis, and to induce apoptosis [19,20,21], previous studies concerning detection and quantitative analysis of MCTs in the indoor environments focused on this class of metabolites [22,23,24]. Furthermore, MCTs were detected in the guttation droplets of *S. chartarum* CT S cultures, which also might contribute to the toxic potential in the indoor environment [25]. Recently, the production of MCTs on different building material was examined by Aleksic et al. [26]. However, in a study from 2002, only one third of *Stachybotrys* isolates, mainly found in water-damaged buildings were attributable to CT S and thus able to produce MCTs [27]. Consequently, approximately two thirds of the identified *Stachybotrys* species mentioned within the study were atranone producers; either *S. chartarum* CT A or *S. chlorohalonata*. Atranones bear a diterpenoid core structure and are known to exhibit less biological activity [28]; additionally, data concerning biological activities are still scarce. Nevertheless, it was reported that *Stachybotrys* isolates consisting of the CT A are linked to strong inflammatory responses in macrophages, which were not exclusively caused by atranones [28], although Rand et al. revealed that, admittedly under appliance of rather high concentrations (2–20 µg/animal), atranones A and C induce dose-and time-dependent inflammatory responses in mice [29]. These findings led to the assumption that other compounds (e.g. PSDs) might be responsible for the observed effects and suggest that the biological effects of the individual metabolites should be investigated rather than complex mixtures. Apart from the mycotoxins, stachylysin, a hemolysin produced by *S. chartarum*, and isolated from the homes of the infants in the previous case, is suspected to contribute to the hemorrhage in infants and irritations of the nasal mucosa in humans [30]. Despite that, the role of the PSDs, which occur in nearly all *Stachybotrys* species and are designated to be its most dominant group among the secondary metabolites, is still unclear. PSDs are meroterpenoids, meaning they are on the one hand derived from the polyketide pathway and on the other hand from the terpene pathway [31]. About 80 metabolites were discovered within this class and a multitude of biological activities was described and reviewed by Wang et al. in 2015 [32]. Notably, one of these metabolites from *Stachybotrys* designated K-76 as well as its derivatives were shown to exhibit inhibitory activities against the complement system and are therefore immunosuppressive agents [33]. This is worth mentioning, as K-76 constitutes a key structure concerning the metabolites, which are dealt with in this study. However, only few investigations concerning the PSDs have been carried out, also due to the limited availability of adequate reference standards, and particularly combinatory effects of the PSDs from *Stachybotrys* have never been considered.

**Figure 1:**
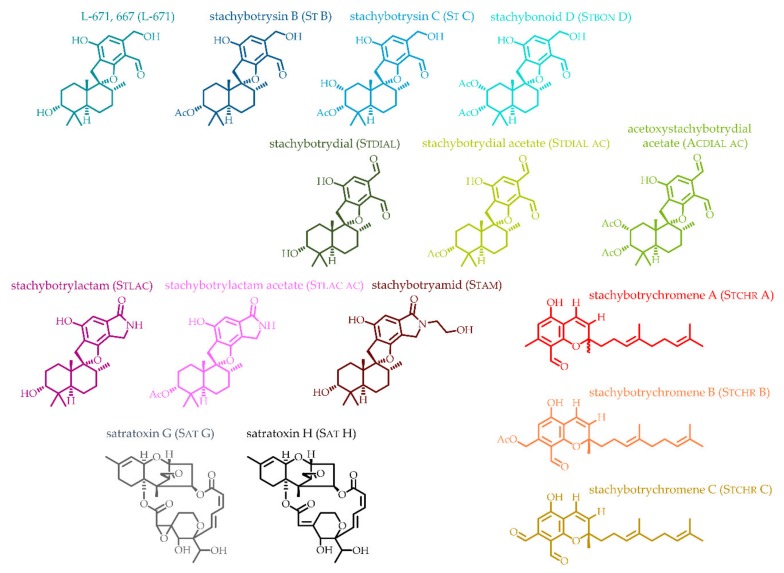
Chemical structures of isolated secondary metabolites from *Stachybotrys* spp. (the used color for each structure is reflected in the chromatograms and all diagrams shown in Figure 2, Figure 3, Figure 4, Figure 5, Figure 6, Figure 7, Figure 8, Figure 9 and Figure 10).

The main objective of the presented study is the quantitative analysis of the most toxicologically important and abundant toxins produced by *Stachybotrys* species. For that purpose, 15 main secondary metabolites (Figure 1) were isolated in order to be used as analytical standards for quantitation by liquid chromatography coupled with tandem mass spectrometry (LC-MS/MS). Different *Stachybotrys* strains were cultivated on various media to examine their characteristic secondary metabolite profile and to compare the strains, especially in terms of their PSD patterns.

## 2. Results

### 2.1. Development and Application of an LC-MS/MS Method for the Detection of Toxins from Stachybotrys

**Figure 2:**
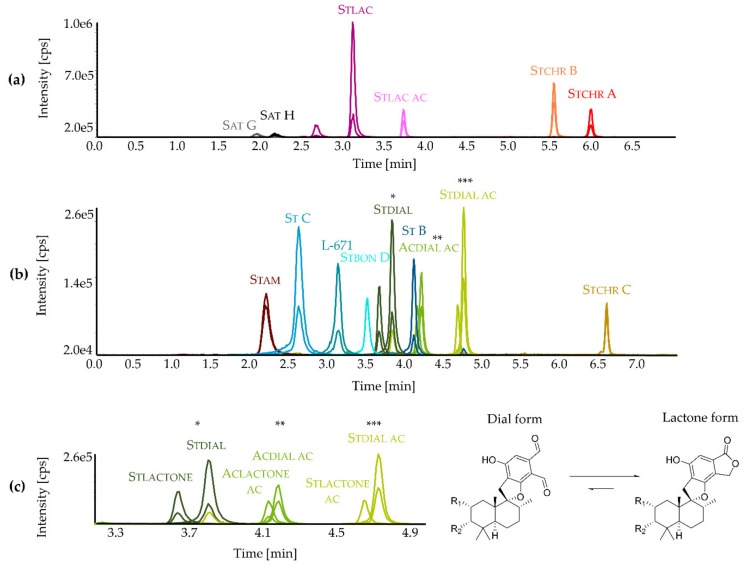
Liquid chromatography coupled with tandem mass spectrometry (LC-MS/MS) chromatograms of isolated secondary metabolites in the positive (**a**) and negative ionization mode (**b**) of a calibration standard with respective selected reaction monitoring (SRM) transitions (details of chromatographic methods are described in Section 5.3); isomerization of dial-containing metabolites to the corresponding lactone forms (**c**) (enlarged view).

Commercial inaccessibility of *Stachybotrys* toxins is a challenging issue and current LC-MS/MS methods for the detection of *Stachybotrys* toxins rely on a semiquantitative approach using the commercially available stachybotrylactam (Stlac) as a reference standard [34]. To overcome this problem, 15 secondary metabolites primarily produced by *Stachybotrys* spp. were isolated and structurally characterized in order to be used as analytical standards to quantitatively analyze the individual metabolic profiles of various strains grown in different media. These 15 reference compounds included the PSDs L-671,776 (L-671), stachybotrysin B (St B), stachybotrysin C (St C), stachybonoid D (Stbon D), stachybotrydial (Stdial), stachybotrydial acetate (Stdial ac), acetoxystachybotrydial acetate (Acdial ac), stachybotrylactam (Stlac), stachybotrylactam acetate (Stlac ac), and stachybotryamide (Stam). They also included the MCTs satratoxin G-H (Sat G, Sat H) as well as the recently discovered stachybotrychromenes A-C (Stchr A-C) [35] (Figure 1). The spectroscopic data (UV, MS, NMR) of all isolated reference compounds can be found in the Supplementary Material Tables S1 and S2. After isolation, an LC-MS/MS method was developed and applied to micro-scale extracts of the fungal cultures grown on agar plates. Figure 2 shows LC-MS/MS chromatograms of a calibration standard mix obtained in the positive (Figure 2a) and in the negative ionization mode (Figure 2b). The dial-containing analytes Stdial, Stdial ac and Acdial ac were observed to undergo isomerization in aqueous solutions, yielding the corresponding lactone forms (Figure 2c). The investigation of the specific mechanism and the corresponding kinetics of this intramolecular Canizzaro reaction [36] were beyond the scope of the presented study and will be addressed in future research.

Interestingly, the developed LC-MS/MS method allows to monitor this isomerization process, as the respective dials and corresponding lactones are chromatographically separated and exhibit distinctive selected reaction monitoring (SRM) transitions. As the ratio of the dial to the lactone form is constantly changing in the samples and the standards, the two forms were quantitated together as a sum parameter and respective concentrations are expressed as dial-containing metabolites in the following sections.

**Figure 3:**
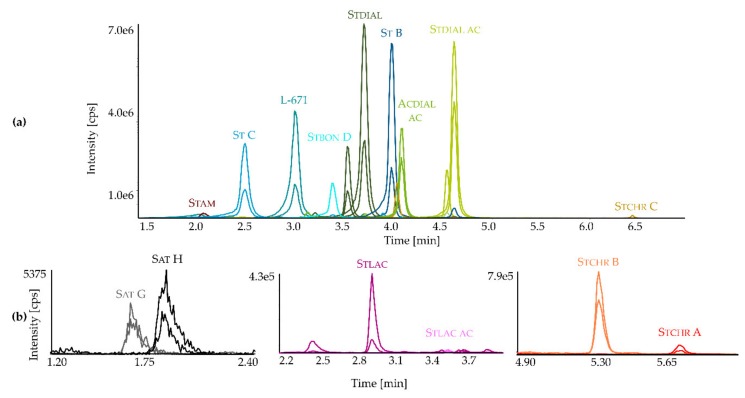
Extracted ion chromatograms with respective SRM) transitions of a micro-scale extract from *S. chartarum* CT S ATCC 34916 after 7 days of growth at 25 °C on potato dextrose agar (PDA) in the dark (1:100 dilution), negative (**a**) and positive ionization mode (**b**) (details of chromatographic methods are described in Section 5.3). The figure is split in several panes, due to the differences in the intensities of the observed metabolites.

Although the PSDs occur in high amounts, a highly sensitive LC-MS/MS method for their detection still offers several advantages. Utilizing the micro-scale extraction method based on Smedsgaard [37] and a subsequent dilute-and-shoot approach enables a simple, fast, and cost-effective solution for the quantitation of *Stachybotrys* toxins in fungal extracts. Despite the employed dilution step, compounds ranking in the lower concentration range (Sat G-H and Stchr A-C) are still detectable. Additionally, chromatographic separation is still sufficient within relatively short run times, even though the PSDs are structurally very similar. Figure 3 exemplarily displays the extracted ion chromatograms with respective SRM transitions in the negative (Figure 3a) and positive ionization mode (Figure 3b) of a micro-scale extract from *S. chartarum* CT S ATCC 34916 after growing for 7 days on potato dextrose agar (PDA) in the dark, whereas all 15 analytes were detectable (corresponding pie chart cf. Supplementary Material Figure S7a).

### 2.2. Secondary Metabolite Profiling of Stachybotrys Strains by LC-MS/MS

Filamentous fungal metabolism depends on several factors, e.g. cultivation conditions and growth media compositions. In order to explore and compare the metabolite profiles of different *Stachybotrys* spp., 5 strains originally isolated from various substrates were selected (Table 1). These strains were cultivated on 4 different media, potato dextrose agar (PDA), synthetic nutrient-poor agar (SNA), malt extract agar (MEA) and Czapek Yeast autolysate agar (CYA). In Section 2.2.1. the biosynthetic production of the two strains—*S. chlorohalonata* vs. *S. chartarum* CT S—is monitored, in Section 2.2.2. the influences of nitrogen(N)-sources on the formation of isoindolinone derivatives are the main focus, and in Section 2.2.3. the secondary metabolite profiles of all strains will be presented. The main results are summarized in pie and column charts, whereas different colors represent different compounds according to Figure 1.

#### 2.2.1. Monitoring of the Biosynthetic Production—*S. chlorohalonata* vs. *S. chartarum* CT S

In order to monitor the biosynthetic production of 15 secondary metabolites by *Stachybotrys* spp., two distinct species were examined. For that purpose, *S. chlorohalonata* and *S. chartarum* CT S were grown on PDA for 21 days, followed by LC-MS/MS analysis of the micro-scale extracts; growth progression and macromorphology of the cultures can be seen in the Supplementary Material Figures S1–S4.

**Figure 4:**
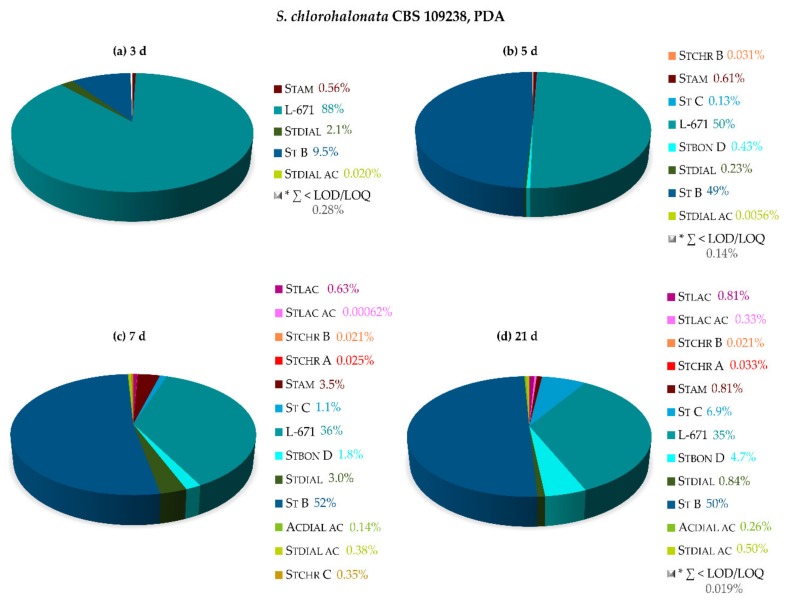
Relative secondary metabolite profiles of *S. chlorohalonata* CBS 109238 on potato dextrose agar (PDA) after 3 days (**a**), 5 days (**b**), 7 days (**c**), and 21 days (**d**) of cultivation at 25 °C in the dark. * ∑ of metabolites < LOD/LOQ (see Figure 1 for chemical structures and abbreviations).

Figure 4 shows the relative metabolite profiles of *S. chlorohalonata* grown on PDA for 3, 5, 7, and 21 days at 25 °C. The metabolite profile after 3 days clearly indicates that L-671 was the first key metabolite (88%), followed by St B (9.5%) that represents an acetylated derivative of L-671. The formation of oxidized L-671 and St B leading to Stdial (2.1%) and Stdial ac (0.020%), respectively, was only slightly visible; the same applied for Stam (0.56%). After 5 days, L-671 and St B were quantitable in roughly comparable amounts (50 and 49%). Moreover, the biosynthesis of further acetylated (Stbon D, 0.43%) as well as hydroxylated metabolites (St C, 0.13%) slightly started and the production of stachybotrychromenes was observed after 5 days of growth. On day 7, an increase in the production of Stdial (3.0%) was observable, corresponding to oxidation of L-671. The proportion of isoindolinone-like compounds slightly increased, whereas Stam (3.5%) constituted the largest proportion of these derivatives. After 21 days of cultivation, a variety of L-671 metabolites primarily contributed to the observed metabolite profile, with St B, St C, and Stbon D, as well as L-671, accounting for approximately 97% of the overall sum of all produced metabolites.

**Figure 5:**
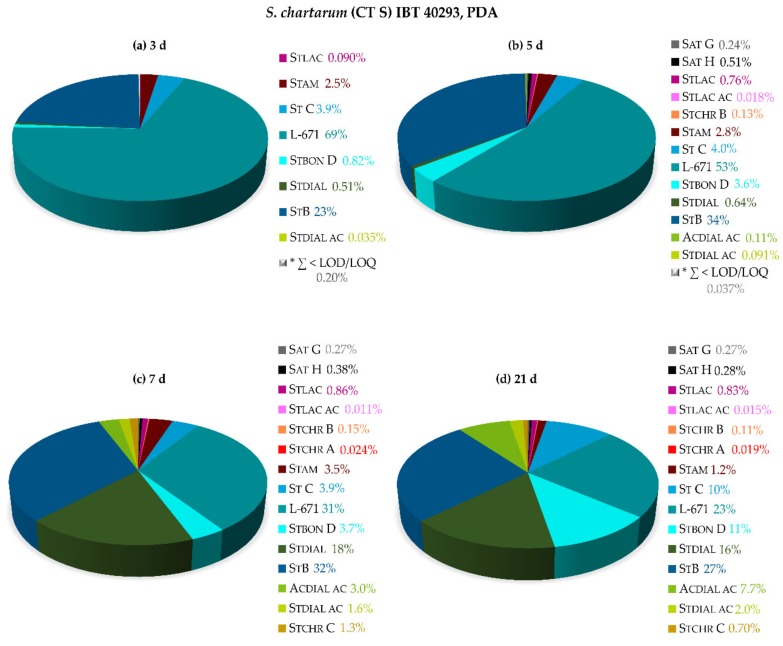
Relative secondary metabolite profiles of *S. chartarum* CT S IBT 40293 on potato dextrose agar (PDA) after 3 days (**a**), 5 days (**b**), 7 days (**c**), and 21 days (**d**) of cultivation at 25 °C in the dark. * ∑ of metabolites < LOD/LOQ (see Figure 1 for chemical structures and abbreviations).

The relative secondary metabolite profiles of *S. chartarum* CT S on PDA after 3, 5, 7, and 21 days of growth at 25 °C are displayed in Figure 5. Initially, the majority of metabolites consisted of L-671 (69%) and St B (23%) after 3 days of growth, in accordance with the profile of *S. chlorohalonata*.

A tendency towards a broader variety of secondary metabolites was, however, visible at this early time point. Especially Stam (2.5%), St C (3.9%), and higher levels of St B (23%) were observed in that regard. Furthermore, satratoxins were determined (Sat G 0.24% and Sat H 0.51%). After one week of growth, all analytes covered by the utilized method were quantitable, illustrating the broad capability of *S. chartarum* CT S to produce these compounds. The vast majority of the total amount of metabolites was attributed to St B (32%), L-671 (31%), and Stdial (18%), followed by St C (3.9%), Stbon D (3.7%), and Stam (3.5%). Levels of further acetylated metabolites of Stdial, namely Stdial ac and Acdial ac, also increased over the course of fungal growth (1.6 and 3.0%). Minor proportions of satratoxins, stachybotrylactams, and -chromenes were determined in the range of 0.011–1.3%. After 21 days of growth, the metabolite profile of *S. chartarum* CT S represented an analogous broad diversity, but with more balanced proportions of St B (27%), L-671 (23%), Stdial (16%), St C (10%), Stbon D (11%), and Acdial ac (7.7%).

**Figure 6:**
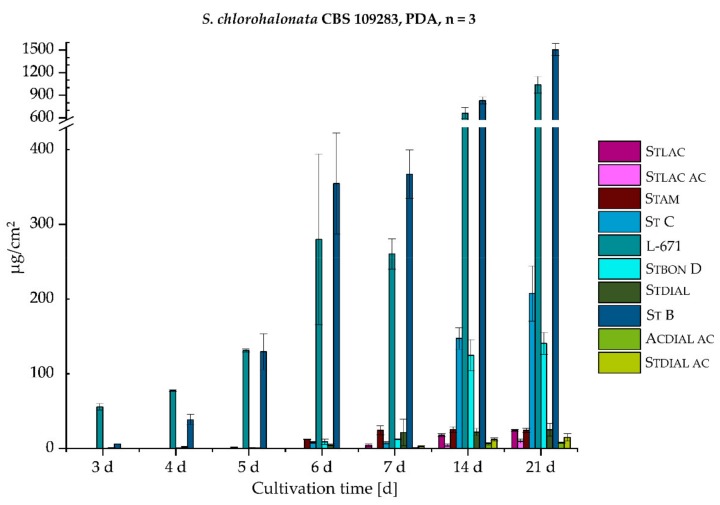
Absolute production of 10 phenylspirodrimanes (PSDs) (µg/cm^2^) by *S. chlorohalonata* over time of growth on PDA) (3–21 days), *n* = 3 (see Figure 1 for chemical structures and abbreviations).

As phenylspirodrimanes (PSDs) represent the most characteristic and prevalent class of secondary metabolites among the genus *Stachybotrys* [38], special focus was put on the versatile PSDs, including recently discovered metabolites, such as St B, St C [39], and Stbon D [40] (later also published as stachybotrysin H [41]). With view to the biosynthetic production of the PSDs and their absolute concentrations in µg/cm^2^, *S. chlorohalonata* and *S. chartarum* CT S were daily sampled over the course of 1 week. Additional sampling points were after 2 and 3 weeks. Figure 6 demonstrates the absolute concentrations of 10 PSDs in µg/cm^2^ produced by *S. chlorohalonata* during 3–21 days. The concentrations of the individual metabolites and the absolute toxin production constantly increased over time.

**Figure 7:**
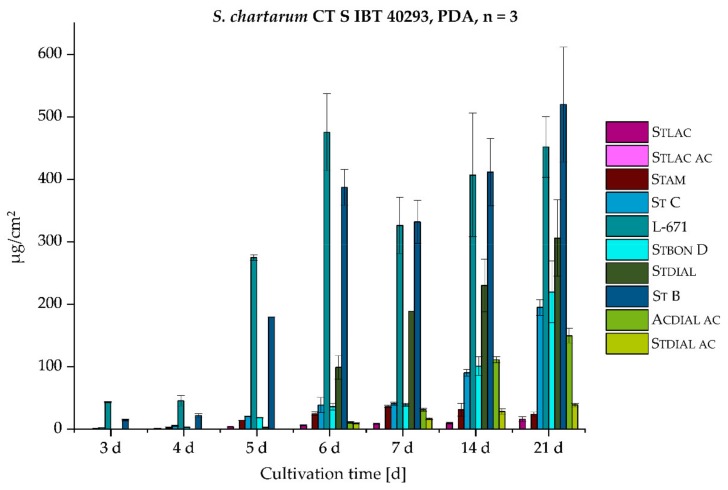
Absolute concentrations of 10 PSDs (µg/cm^2^) by *S. chartarum* CT S over time of growth on PDA (3–21 days), *n* = 3 (see Figure 1 for chemical structures and abbreviations).

In contrast to *S. chlorohalonata* (Figure 6), *S. chartarum* CT S presented a completely different PSD profile (Figure 7), not only in terms of the broader variety of PSDs, but also with regard to the general toxin concentrations. Although *S. chartarum* CT S was able to reach the respective concentrations of *S. chlorohalonata* at earlier time points, the general toxin concentration range after 21 days was only close to two thirds compared to *S. chlorohalonata*. To put this into perspective, the total toxin levels of both strains were compared and are listed in Table 2. On the one hand, after 3 days the total toxin levels of *S. chlorohalonata* steadily increased, and after 21 days a maximum of 3.0 mg/cm^2^ was reached. On the other hand, during the first 6 days total toxin levels of *S. chartarum* CT S slightly increased more rapidly compared to *S. chlorohalonata,* however, reaching a lower total toxin level within the two following weeks (1.9 mg/cm^2^). Nevertheless, the beginning of the PSD biosynthesis (3 and 4 days) was similar to those of *S. chlorohalonata*. The first dominant metabolites of *S. chartarum* CT S were L-671 and St B (day 3 and 4), followed by St C and Stbon D after 5 days. The potential for the formation of further oxidized dial-containing metabolites was higher as well. In addition, the isoindolinone formation (Stam, Stlac, and Stlac ac) was more observed, particularly for Stam. The isoindolinone formation will be dealt with in the following section. Moreover, L-671 and St B roughly reached a plateau after 6 days, while the formation of the oxidized dial-containing metabolites Stdial, Stdial ac, and Acdial ac increased. In summary, *S. chlorohalonata* was capable generally producing higher toxin levels, but with less variety, whereas *S. chartarum* CT S produced a broader variety of metabolites, albeit in lower amounts.

#### 2.2.2. Influence of Nitrogen (N)-Sources and the Formation of Isoindolinone Derivatives

**Figure 8:**
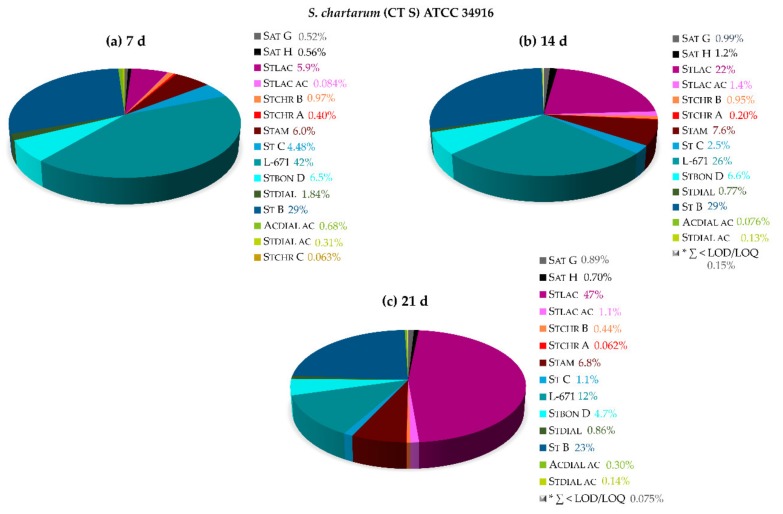
Relative secondary metabolite profiles of *S. chartarum* CT S ATCC 34916 on malt extract agar (MEA) after 7 days (**a**), 14 days (**b**) and after 21 days (**c**) of cultivation at 25 °C in the dark. * ∑ of metabolites < LOD/LOQ (see Figure 1 for chemical structures and abbreviations).

Primary amines can easily perform a nucleophilic attack on *o*-phthalic aldehydes and react to the corresponding imine by the loss of water via the hemiaminal. Hence, it is more than likely that the majority of the PSDs from *Stachybotrys* species is able to undergo this kind of reaction in the presence of amines or ammonia. Thus, the following section revisits this classical condensation reaction to the respective isoindolinones, also referred to as phthalimidines or γ-lactams. Within initial experiments, it turned out that significant levels of Stlac are rarely observable on PDA, whereas a considerable amount of Stlac is detectable when malt extract agar (MEA) is applied. By taking this into account and comparing the total N-content of the individual media, the isoindolinone formation was evaluated. For that purpose, *S. chartarum* (CT S) ATCC 34916 was grown for 21 days on MEA at 25 °C in the dark. Figure 8 displays the influence of the peptone on the metabolite profile, which is typically added to MEA. Peptone comprises peptides and amino acids and was therefore most likely responsible for the increasing Stlac, Stlac ac, and Stam production. Accordingly, the metabolite profile is totally different in comparison to the metabolite profiles on PDA (see Section 2.2.1). On N-poor media, such as PDA and SNA, the relative Stlac proportions were usually <1.5%. Figure 8a evidences that after 7 days of growth Stlac (5.9%) and Stam (6.0%) were generated in higher levels compared to N-poor media. Over the course of the following 14 or 21 days of cultivation, it was clearly discernable that the proportion of Stlac increased over time (22% after 14 days), and after 21 days almost half of the overall amount of produced toxins was attributed to Stlac (47%). After 14 days, Stam (7.6%) and Stlac ac (1.4%) showed the highest levels (Figure 8b).

**Figure 9:**
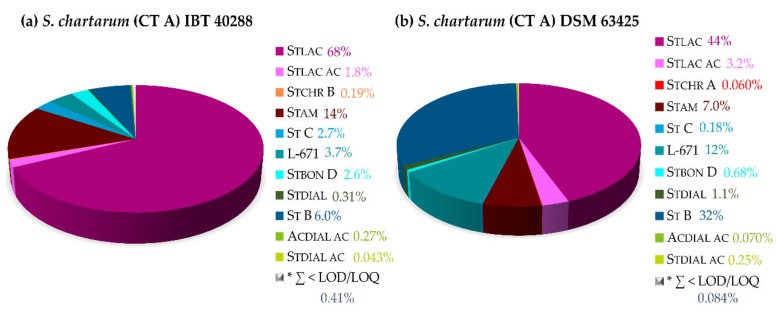
Relative secondary metabolite profiles of *S. chartarum* CT A IBT 40288 (**a**) and *S. chartarum* CT A DSM 63425 (**b**) on Czapek Yeast autolysate agar (CYA) after 21 days of cultivation at 25 °C in the dark (see Figure 1 for chemical structures and abbreviations).

In order to verify and compare the impact of the N-source on the respective metabolite profiles, the investigated strains were cultured on CYA as well. Figure 9 indicates that distinct strains of *S. chartarum* of the same CT generally showed an increased production of isoindolinones on CYA. However, the potential of the strains to form the isoindolinone derivatives is different. The IBT strain produced 68% of Stlac, 1.8% of Stlac ac, and 14% of Stam, whereas the DSM strain produced 44% of Stlac, 3.2% of Stlac ac, and 7.0% of Stam.

#### 2.2.3. Secondary Metabolite Profiles of Various *Stachybotrys* Strains on Synthetic-Nutrient-Poor Agar (SNA)

*Stachybotrys* spp. growth in the indoor environment has been known about for a long time, especially due to the fact that this fungus was firstly isolated from a wallpaper [42]. Thus, in terms of building materials, a medium, which mimics or approximates the nutritional-wise poor conditions, was studied. In this context, SNA was utilized, which only consists of a few salts and very low quantities of sugar. From a macromorphological point of view, the ability of the investigated strains to grow on this medium varied remarkably (Supplementary Material Figure S6). *S. chlorohalonata* and *S. chartarum* CT A (DSM) did not considerably sporulate on SNA after 14 days of cultivation. For *S. chartarum* CT S (IBT) slightly sporulating colonies were observed. On the contrary, *S. chartarum* CT A (IBT) and *S. chartarum* CT S (ATCC) sporulated to a greater extent.

**Figure 10:**
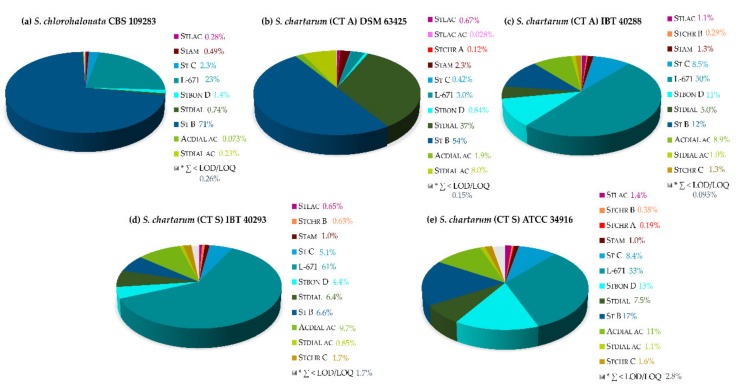
Relative secondary metabolites of 5 different *Stachybotrys* strains after 14 days of cultivation on synthetic-nutrient-poor agar (SNA) at 25 °C in the dark. *S. chlorohalonata* CBS 109283 (**a**), *S. chartarum* CT A DSM 63425 (**b**), *S. chartarum* CT A IBT 40288 (**c**), *S. chartarum* CT S IBT 40293 (**d**) and *S. chartarum* CT S ATCC 34916 (**e**). * ∑ of metabolites < LOD/LOQ (see Figure 1 for chemical structures and abbreviations).

The corresponding metabolite profiles are displayed using pie charts (Figure 10). It becomes evident that *S. chlorohalonata* exhibited the simplest metabolite profile with St B (71%) as the major metabolite, followed by L-671 (23%). Additionally, the general metabolite profile observed on SNA is different from the PDA profile (Figure 4) due to higher relative levels of St B on SNA. The *S. chartarum* CT A strains from DSM strain collection as well as from the IBT collection, differed from *S. chlorohalonata* in terms of their metabolite profiles, but also showed differences among each other. The DSM strain appeared to be the most potent Stdial producer (37%) in comparison with the other strains. Moreover, the DSM strain revealed a considerable production of St B (54%), which was significantly higher in comparison to the other *S. chartarum* strains. *S. chartarum* of CT A and CT S from the IBT collection showed comparable PSD profiles. Apparently, the biosynthesis of *S. chartarum* CT S was different and slower accelerating on SNA than on PDA (Figure 5). 61% of L-671 on SNA after 14 days of cultivation is comparable with the first 3–5 days of cultivation on PDA, but with a broader diversity of further biosynthesized metabolites (primarily St C (5.1%), Stbon D (4.4%), Stdial (6.4%), St B (6.6%), and Acdial ac (9.7%)). *S. chartarum* CT S of the ATCC collection disclosed the most versatile metabolite profile on SNA after 14 days of cultivation, which was roughly comparable to the metabolite profile of *S. chartarum* CT S from the IBT collection cultivated for 21 days on PDA.

Regarding the Stchr A-C biosynthesis, it was apparent that *S. chlorohalonata* showed the lowest individual potential (∑ < LOD/LOQ), followed by *S. chartarum* CT A DSM (Stchr A 0.12%). Only the *S. chartarum* strains from the IBT and ATCC collections (∑ Stchr A-C 1.6–2.3%) are capable producers on the nutrient-poor medium.

Taking the total toxin levels in mg/cm^2^ on PDA into account, the toxin level of *S. chlorohalonata* was approximately 10-fold lower than on SNA; for *S. chartarum* CT S (IBT) it was even approximately 25-fold lower after 14 days (Table 3). Interestingly, the toxin levels of the less sporulating species *S. chlorohalonata* and *S. chartarum* CT A (DSM) were higher than for the other highly sporulating strains, so that there is probably no correlation between the degree of sporulation and the total toxin levels. Particularly in terms of biomass, which were not determinable for *S. chlorohalonata* and *S. chartarum* CT A (DSM) on SNA in this experimental setup, the values are quite high. Bearing in mind that the morphological development and sporulation on building material might be weak as well, these values can be of a particular concern.

## 3. Discussion

The main limitation in assessing the risk of *Stachybotrys* spp. in the indoor environment is the availability of their produced toxins as reference compounds. For this purpose, a series of secondary metabolites occurring in all common *Stachybotrys* species were isolated and structurally characterized in order to be used as reference standards for the development of an LC-MS/MS quantitation method. The latter method enabled the determination of metabolite profiles in micro-scale extracts from fungal cultures by a dilute-and-shoot approach. Within the isolation process, NMR analysis revealed the occurrence of two isomeric forms of the isolated Stdial and its derivatives, namely, their occurrence as a dialdehyde (90%) and the corresponding lactone (10%). The developed LC-MS/MS method allowed for the separate determination of both isomeric forms due to their different chromatographic behavior and mass spectrometric fragmentation. In principle, specific retention time and the neutral loss of CO_2_ from the intact [M+H]^+^ of the lactone during collision-induced dissociation is characteristic. Based on this isomerization and the continuing alteration of the dial/lactone ratio, both isomers were quantitated as a sum parameter. In 1993 Ayer and Miao already reported about the spontaneous transformation of Stdial into the respective γ-lactone [34]. Consequently, another key question is if both isomers exhibit the same toxic or immunosuppressive effects. Distinct mode of actions and biological activities are certainly worth investigating. More details about thermodynamics and kinetics of this isomerization will be addressed in future research.

The class of the phenylspirodrimanes (PSDs), which in turn belong to the class of meroterpenoids, is certainly one of the most dominant and versatile groups of secondary metabolites among the genus *Stachybotrys.* Particularly in recent years, some novel metabolites, such as St B, St C, Stbon D, and triprenyl phenol-like metabolites Stchr A-C, were discovered [35,39,40]. Interestingly, their contribution to the metabolite profiles is noteworthy, not only regarding their role in the biosynthetic pathway, but also to the final metabolite patterns. The characterization of different *Stachybotrys* isolates from water-damaged buildings based on morphology and metabolite production started to be examined in 2002/2003 by Andersen et al. [10,19]. Their findings led to the segregation of *S. chlorohalonata* from *S. chartarum* and they already reported a lower production of PSDs by *S. chlorohalonata* compared to *S. chartarum* [18]. The results obtained in the presented study are in strong agreement with these findings and our results indicate that it may help to potentially distinguish between these two species by LC-MS/MS. Regardless of the chosen media, the metabolic diversity observed for *S. chlorohalonata* was consistently simpler compared to the *S. chartarum* strains. The total toxin levels were however higher compared to *S. chartarum* CT S. One of the first key metabolites within all recorded profiles is always L-671. Regarding this compound, there was a certain amount of confusion concerning the structure and designation. L-671 was firstly isolated in 1992 from a culture of *Memnoniella echinata.* Kaneto et al. isolated in 1994 the so-called Mer-NF 5003E [43]. After structure revision in 1997 by Falck et al., it turned out that L-671 is actually Mer-NF 5003E [44]. Returning to the objective of the comparison of *S. chlorohalonata* and *S. chartarum* CT S, it is plausible that the biosynthetic machinery of *S. chlorohalonata* is distinct or more restricted and that the formation of the oxidized metabolites is not preferred, even after 21 days of cultivation. These findings are remarkably different compared to *S. chartarum* CT S, which showed a broad versatile mixture of metabolites after 21 days of incubation. However, it should be noted that this study only partially covers the whole metabolite spectrum of *Stachybotrys*. Besides the distinction between *S. chartarum* CT A and *S. chlorohalonata* based on its green halo extracellular pigment on CYA [18], comparison of their metabolite profiles might provide an additional tool to distinguish both strains. For that purpose, further *S. chlorohalonata* strains should be examined to validate the metabolite profile observed in this study. The differences in the biosynthesis of the PSDs could also provide an interesting starting point to investigate the biosynthetic machinery of these species in more detail. Regarding the biosynthetic pathway and the results obtained over the course of daily monitoring, the metabolite profiles indicated that formation of the reduced metabolites bearing the drimane modifications, like hydroxylation and acetylation, is favored and has first priority. In contrast, the formation of the oxidized metabolites has second priority for both strains. Furthermore, the production followed the proposed biosynthetic pathways: Sat H is firstly produced, followed by its epoxidation leading to Sat G. Similarly, Stchr A represents the first product in the respective biosynthetic pathway and is subsequently transformed into Stchr B, which in turn is eventually converted to Stchr C after approximately 6 days of fungal growth (cf. Supplementary Material Figure S8).

Stdial and its derivatives Stdial ac and Acdial ac bear an aromatic *o*-dialdehyde moiety, which can easily react with primary amines or generally nitrogen-containing nucleophiles to iso indolinones. Although this reaction has been studied since 1909 [45] and can be applied for the synthesis of heterocyclic compounds [46,47], the complete mechanism is not yet fully elucidated. In 2004, Zuman reviewed the reactions of *o*-phthalaldehyde with nucleophiles and emphasized that information regarding mechanism, kinetics, and equilibria of reactions with *o*-phthalaldehyde and individual nucleophiles is fairly limited [48]. Moreover, reaction products can depend on the suitability of the nucleophile, the reaction environment, and the proportions of the reactants [49]. To further investigate the mechanism, Alajarín et al. computationally studied the condensation reaction and concluded that a three-step-mechanism based on an imine starter unit including an [1,5]-H sigmatropic rearrangement is energetically favored [50]. According to current investigations, the conventionally proposed mechanism via an isoindolinediol intermediate is rather likely due to deuteration experiments [51]. Concerning regioselectivity, D’Hollander and Westwood synthetically examined unsymmetrical *o*-phthalaldehydes with alanine. On the basis of their findings, it can be assumed that the highly regioselective formation of isoindolinone derivatives occur as a result of the position of the aromatic OH-group and are even enhanced by mild reaction conditions [47]. Already in 1995, Jarvis considered whether the stachybotrylactams might be artifacts of the isolation process [38]. Additionally, in 2001, Vértesy et al. isolated memnopeptide A, a terpene peptide, when *M. echinata* was grown on a caseine peptone medium. They suggested that the peptide unit derived from the caseine of the medium was incorporated into the PSD skeleton and serves as an amino storage for the fungus [52]. Particularly in the last years, the number of publications dealing with isoindolinones from *Stachybotrys* species increased [53,54,55]. The specific supply of amino acids to the medium was more precisely investigated by Nishimura et al. (2012) and Yin et al. (2017) [56,57], and their findings are in good agreement with our results. When supplementing the medium with a specific primary amine, the production of the desired metabolite is selectively controlled and enhanced. Returning to the potential bioactivities of the investigated compounds, it should therefore be considered that STDIAL might undergo the described reaction with proteins or other biomolecules bearing accessible amine functions.

MEA and CYA media showed high amounts of Stlac and Stlac ac in our study. MEA is supplemented with soy peptone, whereas CYA comprises yeast extract as N-source. Considering the N-content of the media, CYA has the highest amino-N-content with 0.024–0.032%. In contrast, MEA contains ≥0.013% amino-N and PDA only ≥0.008%. Concerning the total N-content, both media contain almost the same amount of total N ≥0.045%. Therefore, the high amino-N-content of the yeast extract might have a strong impact on the isoindolinone production. Possibly, oxidative deamination of amino acids could take place to ammonia, which afterward reacts with Stdial to form Stlac and Stlac ac. Moreover, high amounts of Stam were observed on both media. Stam is supposed to be the reaction product of Stdial and ethanolamine. Thus, ethanolamine was supplemented to PDA and inoculated with *S. chartarum*. According to the expectations, Stam was selectively generated and led to a simplified isolation process (cf. Section 5.2.3).

None of the used media particularly contained ethanolamine. Therefore, relatively early and constant biosynthesis of Stam on all media might be due to the fact that (mono) ethanolamine plays an essential role in terms of phospholipids, e.g., phosphatidylethanolamine, in biological membranes [58] and thus potentially serves as a source for the isoindolinone unit.

The occurrence of fungi of the genus *Stachybotrys* in the indoor environment has been reported frequently, particularly in water-damaged buildings. Therefore, the metabolite profiles of all 5 strains were investigated on synthetic nutrient-poor agar (SNA) in order to get a rough estimate of the metabolite profile that might be observable on building material, as the latter can be assumed to be similarly poor in nutrients. The results indicated that the different strains possess an individual metabolite profile. Apparently, the most potent strain among the examined is the ATCC strain *S. chartarum* CT S. Remarkably, this strain was involved in stachybotrytoxicosis cases in Hungary, whereby mostly the satratoxins were considered to be responsible [13]. In these cases, it was reported that straw and feedstuffs were frequently contaminated with *Stachybotrys* and led to diseases like irritation of the skin, dermal necrosis, hemorrhage, nervous disorder, and even death of farm livestock [14,59]. Taking together these facts, they provide further hints that the combination and broad variety of the *Stachybotrys* toxins, including the highly toxic satratoxins, caused the described symptoms. Future research should clearly address combinatory effects of all these toxins, as previous studies focused primarily only on satratoxins.

Interestingly, low proportions of Stlac could still be detected when the fungus was grown on SNA, even though this medium did not contain any N-source besides KNO_3_. Yin et al., however, proved the occurrence of nitrate reductase genes in the genomes of *S. chlorohalonata* and *S. chartarum* [54]. The nitrate present in the medium might thus have been enzymatically converted to ammonia [60], which underwent reaction with Stdial to yield Stlac.

A culture filtrate of *S. complementi* nov. sp. K-76 showed strong inhibition towards the complement system, hence the isolated compound was named K-76 [61]. K-76 is a Stdial derivative possessing a second hydroxy group attached to the C-2 of the drimane scaffold. As K-76 is described to inhibit the complement system and therefore acts as an immunosuppressive reagent, it is reasonable to assume that other analogs could reveal similar properties. In recent years, many other biological activities were reported [32,33,62,63,64] and demonstrate the exceptional role of these secondary metabolites among the genus *Stachybotrys*.

## 4. Conclusions

The present study was conducted to gather deeper insights into the individual potentials of *Stachybotrys* spp. primarily found in the indoor environment to biosynthesize phenylspirodrimanes (PSDs). This is the first study dealing with such a multitude of compounds in a quantitative manner and monitoring their production on several media. For that purpose, 15 characteristic metabolites were isolated from cultures of *Stachybotrys* spp. as analytical standards. Two species frequently found in water-damaged buildings, namely, *S. chlorohalonata* and *S. chartarum* CT S, were compared regarding their potential to produce PSDs. It was observed that they exhibited distinctive patterns in terms of the production of PSDs. *S. chlorohalonata* was capable to generally produce higher toxin amounts but with less variety, whereas *S. chartarum* CT S produced a broader variety, albeit in lower amounts. Principally, it was possible to monitor the biosynthetic pathway of the respective metabolites. In addition, the developed LC-MS/MS multimethod enabled the accurate observation of the dial/lactone isomerization, which spontaneously takes place in aqueous solutions over the course of time. The formation of isoindolinone derivatives is favored on N-rich media due to a simple non-enzymatic reaction of dialdehydes with ammonia, amino acids, or primary amines. Even though the reaction has been known for a long time, the mechanism is still not fully understood. Taking this into account, Stlac is an artifact rather than a genuine marker for *Stachybotrys* growth, and other metabolites occurring on all media in considerable amounts should rather be considered. The metabolite production on the nutrient-poor SNA revealed lower concentrations, however, in particular, *S. chartarum* strains exhibited proper growth and accordingly offered a broad metabolite spectrum. Generally, the examined different strains, even of the same species and CT, featured distinct compositions of PSDs, which offers quite an interesting potential for future investigations. The metabolite profiles of *Stachybotrys* species consequently depend on time, substrate, and the individual biosynthetic potential of the respective strain. Thus, the analysis of real samples from *Stachybotrys* infested building material provides valuable insights into which strains and metabolites are of relevance. Combinatory effects of the metabolites should also be considered in future research to clarify the human health risks concerning *Stachybotrys* growth in the indoor environments.

## 5. Materials and Methods

### 5.1. Fungal Strains and Media Compositions

*S. chartarum* IBT 40293, *S. chartarum* IBT 40288 and *S. chartarum* ATCC 34916 were kindly provided by M. Gareis (Ludwig-Maximilians-Universität München, Germany). *S. chartarum* DSM 63425 was purchased from Leibniz Institute DSMZ-German Collection of Microorganisms and Cell Cultures (DSMZ, Braunschweig, Germany). *S. chlorohalonata* CBS 109283 was from the Westerdijk Fungal Biodiversity Centre (CBS, Utrecht, The Netherlands). After reviving the freeze-dried-cultures, they were maintained on slants of potato dextrose agar (PDA, ready-to-use medium, 4 g/L potato extract, 20 g/L dextrose, 15 g/L agar, Sigma-Aldrich, Steinheim, Germany) medium at 4 °C. Media were autoclaved at 121 °C for 15 min. For highly sporulating pre-cultures, the fungal strains were grown on Petri dishes (92 × 16 mm, Sarstedt, Nümbrecht, Germany) with PDA medium for 14 days at 25 °C in the dark. Spore suspensions were prepared from these cultures by adding 10 mL of sterile water (containing 0.05% Tween 80) to the agar plate and the number of spores was determined using a Neubauer cell chamber. Afterward, the spore suspensions were diluted to the required concentrations of 10^6^ spores/mL. Then, 100 µL spore suspension of each strain was applied by 3-point-inoculation on the respective medium on agar plates and cultured at 25 °C in the dark at saturated humidity. The following media compositions were used: malt extract agar (MEA, pH 5.4, 30 g/L malt extract (Roth, Karlsruhe, Germany), 5 g/L (soy) peptone (Roth, Karlsruhe, Germany), 15 g/L agar-agar Kobe I (Roth, Karlsruhe, Germany), Czapek yeast autolysate agar (CYA, pH 7.3, sucrose 30 g/L (VWR, Darmstadt, Germany), NaNO_3_ 3 g/L (Roth, Karlsruhe, Germany), K_2_HPO_4_ 1 g/L (VWR, Darmstadt, Germany), MgSO_4_ × 7H_2_O 0.5 g/L (VWR, Darmstadt, Germany), KCl 0.5 g/L (Roth, Karslruhe, Germany), FeSO_4_ × 7H_2_O 0.01 g/L (VWR, Darmstadt, Germany), yeast extract 5 g/L (Roth, Karlsruhe, Germany); 15 g agar-agar Kobe I (Roth, Karlsruhe, Germany), synthetic-nutrient-poor agar (SNA, pH 5.4, KH_2_PO_4_ 1 g/L (VWR, Darmstadt, Germany), KNO_3_ 1 g/L (Roth, Karlsruhe, Germany), MgSO_4_ × 7H_2_O 0.5 g/L, KCl 0.5 g/L, glucose 0.2 g/L (VWR, Darmstadt, Germany), sucrose 0.2 g/L (VWR, Darmstadt, Germany), agar-agar Kobe I 15 g/L (Roth, Karlsruhe, Germany). The colony areas (cm^2^) of the cultures (Supplementary Material Figures S1–S3) were determined by measuring 3 colonies from 3 independent agar plates, and afterward the average value was formed.

### 5.2. Isolation and Analytical Data of 15 Stachybotrys Toxins

#### 5.2.1. Isolation of Sat G and Sat H

Initially, a liquid pre-culture of *S. chartarum* CBS 109291 CT S in an Erlenmeyer flask containing 200 mL of potato dextrose broth (Roth, Karlsruhe, Germany) was incubated for 4 days at 25 °C in the dark on a laboratory shaker (85 rpm). 28 baby food jars were filled with 20 g rice (long grain rice Uncle Ben’s^®^) each and 10 mL aqueous glucose-solution (glucose 38 g/L) was added per glass. The rice was allowed to stand at room temperature for 3 days to swell. Afterward, the glasses with rice were autoclaved (15 min, 121 °C), and after cooling down, inoculated with 10 mL of the pre-culture. The rice cultures were grown in the dark at 25 °C. After 3 weeks, the rice was extracted with ethyl acetate for 30 min by sonification and 60 min on a laboratory shaker (300 rpm). The extract was filtered through Miracloth (Merck, Darmstadt, Germany) and the extraction step was repeated twice, after which the extracts were combined and evaporated at 40 °C on a rotary evaporator to dryness. The residue was dissolved in dichloromethane and applied to manual normal phase column chromatography (column size 30 × 3 cm, silica gel 60 (0.040–0.063 mm, Merck, Darmstadt, Germany) for purification and fractionation by using a cyclohexane/ethyl acetate gradient (9:1, 7:3, 1:1, 3:7, 0:100 (*v/v*)). The solvent ratio was changed based on thin-layer chromatography analysis of the fractions. Fractions, containing satratoxins, were combined and concentrated to dryness on a rotary evaporator at 40 °C. Further purification and isolation of SAT G and H was maintained by semi-preparative HPLC-UV (LC-NetII/ADC Jasco Labor und Datentechnik, Gross-Umstadt, Germany). For that purpose, a Lichrosorb^®^ RP-18 column (250 × 10 mm, 7 µm; Merck, Darmstadt, Germany) was used with a flow rate of 5 mL/min and isocratic elution of water/methanol (45/55; *v/v*) and UV detection at a wavelength of 255 nm. After this step, SAT G was obtained with sufficient purity, whereas the purity of SAT H was insufficient. Therefore, a second semi-preparative approach was conducted by using an Agilent Eclipse XDB-C18 column (250 × 9.4 mm, 5 µm; Waldbronn, Deutschland) with a flow rate of 5 mL/min and isocratic elution of water/methanol (45/55; *v/v*) and UV detection at a wavelength of 225 nm. SAT G (11 mg) and H (10 mg) were obtained as white amorphous solids with purities ≥95% (based on HPLC-DAD-ELSD).

#### 5.2.2. Isolation of St B, St C, L-671, Stbon D, Stdial, Stdial ac, and Acdial ac

*S. chartarum* CT A IBT 40288 was cultivated on PDA and after 21 days of incubation at 25 °C in the dark agar plates were extensively extracted with ethyl acetate, meaning sonication for 30 min and shaking on a laboratory shaker for 60 min at 230 rpm. Then, the extract was filtered through Miracloth filter material to remove the remaining spores. The extraction procedure was repeated twice, and the extracts were combined and evaporated to dryness at 40 °C. Thereafter, the obtained crude residue was pre-purified and fractionated by manual normal phase column chromatography (column size 30 × 3 cm, silica gel 60, 0.04–0.063 mm, Merck, Darmstadt, Germany) using a cyclohexane/ethyl acetate gradient (9:1, 7:3, 1:1, 3:7, 0:100 (*v/v*)). The solvent ratio was changed based on thin-layer chromatography analysis of the fractions. Fractions containing the respective metabolite/s were combined. Subsequently, separation and purification were carried out on a Jasco semi-preparative HPLC system (Section 5.2.1). In the following, semi-preparative parameters (column; flow rate; elution; detection wavelength) are listed for each metabolite:

- St B: Reprosil-Pur 120 C18-AQ (250 × 10 mm, 5 μm; Dr. Maisch GmbH, Ammerbuch, Germany); 3 mL/min; isocratic elution, water/methanol (15/85; *v/v*); 254 nm.
- St C and L-671: Agilent Eclipse XDB-C18 column (250 × 9.4 mm, 5 µm; Waldbronn, Germany); 4 mL/min, isocratic elution water/MeCN (50/50; *v/v*); 360 nm.
- Stbon D and Stdial ac: Agilent Eclipse XDB-C18 column (250 × 9.4 mm, 5 µm; Waldbronn, Germany); 4 mL/min, gradient elution starting from 50% MeCN in 20 min to 100% MeCN; 254 nm.
- Stdial and Acdial ac: Agilent Eclipse XDB-C18 column (250 × 9.4 mm, 5 µm; Waldbronn, Germany); 4 mL/min, gradient elution starting from 60% MeCN in 15 min to 100% MeCN; 254 nm.

#### 5.2.3. Isolation of Stlac, Stlac ac, and Stam

Stlac and Stlac ac were isolated by the cultivation of *S. chartarum* CT A IBT 40288 on MEA and after 21 days of incubation at 25 °C in the dark, agar plates were extensively extracted with ethyl acetate. The further extraction and column chromatography procedure was analogous to Section 5.2.2. Semi-preparative HPLC-UV was carried out using a Reprosil-Pur 120 C18-AQ column (250 × 10 mm, 5 μm; Dr. Maisch GmbH, Ammerbuch, Germany) at a flow rate of 5 mL/min with isocratic elution of water/MeCN (60/40; *v/v*) at 300 nm.

Stam was isolated from *S. chartarum* CT A DSM 63425. Therefore, PDA was supplemented with 20 mM ethanolamine after autoclaving and inoculated with the fungal culture. This approach allowed to selectively isolate the desired metabolite in a sufficient amount. The extraction procedure was analogous as described above, whereas the column chromatography step was no longer mandatory. Finally, semi-preparative HPLC-UV for purification Reprosil-Pur 120 C18-AQ column (250 × 10 mm, 5 μm; Dr. Maisch GmbH, Ammerbuch, Germany) at a flow rate of 5 mL/min with isocratic elution of water/MeCN (60/40; *v/v*) at 300 nm was used.

#### 5.2.4. Isolation of Stchr A-C

Stchr A-C were isolated according to Jagels et al. 2018 [35].

#### 5.2.5. Acquirement of Analytical Data of All 15 Reference Standards

For the determination of accurate masses (Supplementary Material Table S1) of isolated references, an LTQ Orbitrap XL (Thermo Fischer Scientific, Walthma, MA, USA) was used with heated electrospray ionization in positive and negative mode. Sample solutions were directly applied via syringe pump. The capillary temperature was set to 275 °C and the source voltage was 4.0 kV, capillary voltage was 40 V and Tube Lens was 130 V. Sheath gas flow was 5 arbitrary units, whereas neither auxiliary gas and sweep gas were applied nor a source temperature set. UV spectra were obtained with a J-750 Spectrophotometer (Jasco, Groß-Umstadt, Germany) in MeCN. All isolated reference compounds were of purity ≥95% (HPLC-DAD-ELSD, Shimadzu, Kyoto, Japan). NMR data are presented in Supplementary Material Table S2, ^1^H, ^13^C, gCOSY, gHSQC, and gHMBC spectra were recorded on an Agilent DD2 600 MHz spectrometer (Santa Clara, CA, USA); δ in ppm related to the solvent/tetramethylsilane).

### 5.3. Sample Preparation and Method Performance of 15 Stachybotrys Toxins

For extraction, a slightly modified micro-scale extraction method developed by Smedsgaard was used [37]. Three plugs (1.3 cm^2^) from three different agar plates were cut from the fungal colonies with a sterile corer. In case of colonies <1.3 cm^2^, the entire colony was cut out, whereby the colony area was previously determined (Section 5.2.5 and Supplementary Material Figures S1–S3). The agar plug was transferred to a 2 mL reaction tube (Eppendorf, Hamburg, Germany) and 1 mL of MeCN. was added. Subsequent extraction was carried out by an ultrasonic bath (Bandelin Sonorex^TM^ RK 100, Berlin, Germany) for 30 min. Afterward, the supernatant was removed, and extraction was repeated. The supernatants were combined, centrifuged for 5 min at 15,000 xg, and diluted to the required concentration for LC-MS/MS analysis.

LC-MS/MS analysis of all references and sample extracts was carried out by an Agilent 1260 Infinity series HPLC (Agilent Technologies) coupled to a QTrap® 5500 mass spectrometer with an electrospray ionization (ESI) source (Sciex, Darmstadt, Germany). For data acquisition and subsequent analysis, the Analyst^®^ software (v1.6.2, Sciex) was used. All chromatograms were smoothed with a smoothing width of 3 points. The mass spectrometric parameters, as well as parent and fragment ions (quantifier and qualifier) for all analytes, were optimized by syringe pump infusion in MeCN/water (+0.1% formic acid (FA)) (1:1, *v/v*).

Chromatography was performed using a Nucleodur^®^ C_18_ Gravity-SB column (100 × 2 mm, 3 μm particle size, Macherey Nagel, Düren, Germany) attached with a guard column (4 × 2 mm) of the same material. Column oven temperature was set to 40 °C and the sample injection volume was 5 μL. A binary gradient consisting of MeCN (eluent A) and water (eluent B) (both +0.1% FA) at a constant flow rate of 500 μL/min was used. For the compounds analyzed in the positive mode, a gradient was applied as following: 0.00 min, 45% A; 0.50 min, 45% A; 3.00 min, 97.5% A; 5.50 min, 97.5% A; 5.51 min, 45% A; 7.00 min, 45% A. For the negative mode, starting conditions were 50% of A held for 0.5 min and increased up to 97.5% in 4.5 min. A was then constantly held until 6.0 min and at 6.01 min decreased to 50% A until 7.5 min. The setting parameters for positive/negative ionization modes were applied as following: ionization voltage, 4.5/−4.5 kV; curtain gas, 35 psi; nebulizer gas, 35 psi; heater gas, 45 psi; source temperature 500 °C; entrance potential, 10/−10 V and cell exit potential 10/−10 V. Unit resolution was applied and the collision gas was set to “high”. Declustering potential (DP), as well as collision energy voltage (CE), were optimized for each analyte ([M+H]^+^, [M+Na]^+^, [M-H]^−^) by direct infusion using the syringe pump (Supplementary Material Table S3). The respective SRM transitions were recorded with a dwell time of 25 ms (negative mode) and 40 ms (positive mode).

The determination of the limit of detections (LODs) and limit of quantitations (LOQs) was performed by dilution of the respective reference standard in starting conditions of the HPLC. The signal to noise ratio (S/N) of 3 was considered for the LODs and S/N ≥10 for the LOQs of the qualifier. Starting from the stock solutions (kept at −20 °C in the dark before the measurements), 8 calibration solutions were equidistantly prepared in the range between 2 decades for each analyte by dilution with the mobile phase of the starting composition of the HPLC run (Supplementary Material Table S4). All analytes were absolutely quantitated (ng/cm^2^) and afterward summed and set to 100%, with the not detectable and quantifiable analytes (∑ < LOD/LOQ) included with their highest values as well. Then, for each analyte and the analytes ∑ < LOD/LOQ the percentage yields were calculated, representing relative amounts. Recovery rates, rather defined as matrix-interference values, were in the range of 76–129% (lowest dilution).

## Figures and Tables

**Table 1:** Examined *Stachybotrys* strains with corresponding origin/substrate type and collection identity number.

Species	Origin/Substrate	Collection
*S. chlorohalonata* ^a^	Denmark/building material	CBS 109283 ^c^
*S. chartarum*^b^ CT A	California/building material	IBT 40288 ^d^
*S. chartarum*^b^ CT S	California/building material	IBT 40293 ^d^
*S. chartarum*^b^ CT A	Nepal/soil	DSM 63425 ^e^
*S. chartarum*^b^ CT S	Hungary/oats	ATCC 34916 ^f^

^a^ B Andersen & Thrane; ^b^ (Ehrenberg) Hughes; ^c^ CBS: Culture collection in Utrecht, the Netherlands (cf. IBT 9293); ^d^ IBT: Culture collection at BioCentrum-DTU, Denmark; ^e^ DSM: Leibniz Institute DMZ-German Collection of Microorganisms and Cell Cultures; ^f^ ATCC: American Type Culture Collection.

**Table 2:** Total toxin levels in mg/cm^2^ of 15 *Stachybotrys* metabolites produced by *S. chlorohalonata* and *S. chartarum* CT S grown on potato dextrose agar (PDA) after 3–21 days.

	*S. chlorohalonata* CBS 109283	*S. chartarum* CT S IBT 40293
Day	Toxin Level [mg/cm^2^]	Toxin Level [mg/cm^2^]
3	0.0063	0.063
4	0.12	0.088
5	0.27	0.52
6	0.67	1.1
7	0.73	1.0
14	1.9	1.4
21	3.0	1.9

**Table 3:** Total toxin levels in mg/cm^2^ of 15 *Stachybotrys* metabolites produced by the examined strains on synthetic nutrient-poor agar (SNA) after 14 days of cultivation.

14 Days	*S. chlorohalonata* CBS 109283	*S. chartarum* CT A DSM 63425	*S. chartarum* CT A IBT 40288	*S. chartarum* CT S IBT 40293	*S. chartarum* CT S ATCC 34916
Toxin level [mg/cm^2^]	0.17	0.17	0.065	0.053	0.089

## Supplementary Materials

The following are available online at https://www.mdpi.com/2072-6651/11/3/133/s1, Table S1: Analytical data of isolated *Stachybotrys* secondary metabolites, Table S2: NMR data of isolated *Stachybotrys* secondary metabolites, Table S3: SRM parameters for all 15 analytes (tR retention time, DP declustering potential, CE collision energy, Table S4: LODs, LOQs, and working ranges in ng/mL of the 15 *Stachybotrys* analytes, Figure S1: Colony areas in cm^2^ of *S. chlorohalonata* CBS 109283 and *S. chartarum* CT S IBT 40293 grown on PDA (time 3, 4, 5, 6, 7, 14, and 21 days), Figure S2: Colony areas in cm^2^ of *S. chartarum* (CT S) IBT 40293 grown on PDA, *S. chartarum* (CT S) ATCC 34916 grown on MEA and *S. chartarum* (CT A) IBT 40288 and *S. chartarum* (CT A) DSM 63425 grown on CYA (time 7, 14, and 21 days), Colony areas in cm^2^ of *S. chlorohalonata* CBS 109283, *S. chartarum* CT A IBT 40288, *S. chartarum* CT A DSM 63425, *S. chartarum* CT S ATCC 34916 grown on MEA and *S. chartarum* CT S IBT 40293 grown on SNA (time 7, 14, and 21 days), Figure S3: Colony areas in cm^2^ of *S. chlorohalonata* CBS 109283, *S. chartarum* CT A IBT 40288, *S. chartarum* CT A DSM 63425, *S. chartarum* CT S ATCC 34916 grown on MEA and *S. chartarum* CT S IBT 40293 grown on SNA (time 7, 14, and 21 days), Figure S4: Macromorphology of *S. chlorohalonata* CBS 109283 (a) and *S. chartarum* (CT S) IBT 40293 (b) on PDA after 3, 5, 7, and 21 days of cultivation at 25 °C in the dark, Figure S5: Macromorphology of *S. chartarum* (CT S) ATCC 34916 on MEA after 7 days (a), 14 days (b) and 21 days (c) of cultivation at 25 °C in the dark, Figure S6: Macromorphology of *S. chlorohalonata* CBS 109283 (a), *S. chartarum* (CT A) DSM 63425 (b), *S. chartarum* (CT A) IBT 40288 (c), *S. chartarum* (CT S) IBT 40293 (d) and *S. chartarum* (CT S) ATCC 34916 (e) on SNA after 14 days of cultivation at 25 °C in the dark, Figure S7: Relative secondary metabolite profiles of *S. chartarum* CT S ATCC 34916 on PDA after 7 days (a), 14 days (b) and 21 days (c) of cultivation at 25 °C in the dark, Figure S8: Proposed biosynthetic pathways of secondary metabolites produced by *Stachybotrys* spp.
