# Supplementary material for: Exploring Secondary Metabolite Profiles of Stachybotrys spp. by LC-MS/MS
Source: Toxins (Basel). 2019 Feb 27;11(3):133. doi: 10.3390/toxins11030133 (PMC6468463; doi:10.3390/toxins11030133)
Supplement: Supplementary file 1 [file toxins-11-00133-s001.pdf]

toxins

MDPI

Supplementary Material

Exploring Secondary Metabolite Profiles of *Stachybotrys* spp. by LC-MS/MS

1. Analytical and Spectroscopic Data

Table S1. Analytical data of isolated *Stachybotrys* secondary metabolites*.

Compound	Molecular Formula	Appearance	UV $\lambda_{max.}$ nm (ACN)	Accurate mass (Δ ppm)
SAT G	$C_{29}H_{36}O_{10}$	White amorphous solid	225, 255	545.2386 (+0.9) [M + H]$^+$
SAT H	$C_{29}H_{36}O_9$	White amorphous solid	225, 238, 265	529.2433 (+0.2) [M + H]$^+$
STLAC	$C_{23}H_{31}NO_4$	White amorphous solid	220, 260, 300	386.2304 (-5) [M + H]$^+$
STLAC AC	$C_{25}H_{33}NO_5$	White amorphous solid	220, 260, 298	428.2432 (+0.2) [M + H]$^+$
STCHR B	$C_{25}H_{32}O_5$	Brownish oil	211, 278, 325, 363	411.2177 (0.0) [M-H]$^-$
STCHR A	$C_{23}H_{30}O_3$	Brownish oil	211, 278, 321, 363	353.2115 (−2) [M-H]$^-$
STAM	$C_{25}H_{35}NO_5$	Pale yellow amorphous solid	220, 262, 298	430.2559 (−7) [M + H]$^+$
ST C	$C_{25}H_{34}O_7$	Pale yellow amorphous solid	226, 240, 286, 328	447.2368 (−2) [M + H]$^+$
L-671	$C_{23}H_{32}O_5$	Pale yellow amorphous solid	228, 242, 286, 328	389.2321 (−0.5) [M + H]$^+$
STBON D	$C_{27}H_{36}O_8$	Pale yellow amorphous solid	226, 240, 286, 328	489.2477 (−1) [M + H]$^+$
STDIAL	$C_{23}H_{30}O_5$	Pale white amorphous solid	220, 240, 308, 354	387.2160 (+1) [M+H]$^+$
ST B	$C_{25}H_{34}O_6$	White amorphous solid	228, 240, 286, 328	431.2427 (−0.2) [M + H]$^+$
ACDIAL AC	$C_{27}H_{34}O_8$	White amorphous solid	220, 240, 304, 350	487.2325 (−0.2) [M + H]$^+$
STDIAL AC	$C_{25}H_{32}O_6$	White amorphous solid	200, 254, 306, 354	429.2269 (−0.7) [M + H]$^+$
STCHR C	$C_{23}H_{28}O_4$	Yellow oil	211, 282, 365	367.1915 (0.0) [M-H]$^-$

*Purity ≥95% (HPLC-DAD-ELSD).

Table S2. NMR data of isolated *Stachybotrys* secondary metabolites.

^{1}H NMR	^{13}C NMR
**SAT G (CDCl$_3$)**	
δ [ppm] = 6.97 (dd, J = 16.8, 8.0 Hz, 1H, H-8′), 6.68 (dd, J = 10.8, 8.0 Hz, 1H, H-9′), 5.89 (m, 3H, H-7′). 5.44 (d, J = 5.2 Hz, 1H, H-10), 4.38 (m, 2H, H-13′), 4.13 (d, J = 12.7 Hz, 1H, H-15), 4.00 (m, 1H, H-5′), 3.94 (m, 1H, H-5′), 3.86 (d, J = 4.9 Hz, 1H, H-2), 3.61 (d, J = 5.3 Hz, 1H, H-11), 3.44 (s, 1H, H-2′), 3.29 (s, 1H, H-12′), 3.15 (d, J = 4.0 Hz, 1H, H-13), 2.84 (d, J = 3.9 Hz, 1H, H-13), 2.47 (m, 4H, H-3), 2.23 (m, 1H, H-4′), 2.04 (m, 1H, H-8), 1.90 (m, 2H, H-7), 1.73 (s, 3H, H-16), 1.12 (d, J = 6.5 Hz, 3H, H-14′), 0.86 (s, 3H, H-14).	δ [ppm] = 167.0 (1C, C-11′), 166.9 (1C, C-1′), 144.2 (1C, C-9′), 140.4 (1C, C-9), 131.8 (1C, C-7′), 131.5 (1C, C-8′), 119.9 (1C, C-10′), 118.6 (1C, C-10), 81.4 (1C, C-6′), 79.2 (1C, C-2), 73.7 (1C, C-4), 72.4 (1C, C-12′), 70.0 (1C, C-13′), 68.1 (1C, C-11), 65.4 (2C, C-12, C-3′), 64.9 (1C, C-15), 61.0 (1C, C-2′), 60.1 (1C, C-5′), 49.2 (1C, C-5), 48.1 (1C, C-13), 43.2 (1C, C-6), 34.3 (1C, C-3), 27.5 (1C, C-8), 23.4 (1C, C-16), 22.6 (1C, C-4′), 20.1 (1C, C-7), 16.0 (1C, C-14′), 8.0 (1C, C-14).
**SATH (CDCl$_3$)**	
δ [ppm] = 7.34 (dd, J = 17.2, 10.1 Hz, 1H, H-8′), 6.61 (t, J = 10.1 Hz, 1H, H-9′), 6.08 (d, J = 17.0 Hz. 1H, H-7′), 5.90 (m, 2H, H-10′), 5.84 (bs, 1H, H-2′), 5.45 (d, J = 5.1 Hz, 1H, H-10), 4.56 (d, J = 12.6 Hz, 1H, H-15), 4.35 (q, J = 6.5 Hz, 1H, H-13′), 3.99 (s, 1H, H-12′), 3.87 (m, 4H, H-4′, H-5′, H-15), 3.75 (dt, J = 10.7, 2.9 Hz, 1H, H-5′), 3.60 (d, J = 5.3 Hz, 1H, H-11), 3.14 (d, J = 4.1 Hz, 1H, H-13), 2.82 (d, J = 4.1 Hz, 1H, H-13), 2.65 (m, 1H, H-4′), 2.44 (dd, J = 15.2, 8.5 Hz, 1H, H-3), 2.20 (m, 1H, H-3), 2.05 (m, 2H, H-8), 1.92 (m, 2H, H-7), 1.73 (s, 3H, H-16), 1.16 (d, J = 6.6 Hz, 3H, H-14′), 0.82 (s, 3H, H-14).	δ [ppm] = 167.1 (1C, C-11′), 166.2 (1C, C-1′), 154.9 (1C, C-3′), 142.9 (1C, C-9′), 140.4 (1C, C-9), 134.3 (1C, C-8′), 132.0 (1C, C-7′), 120.4 (1C, C-10′), 119.1 (1C, C-2′), 118.9 (1C, C-10), 81.3 (1C, C-6′), 79.1 (1C, C-2), 74.1 (1C, C-4), 73.6 (1C, C-12′), 69.7 (1C, C-13′), 68.1 (1C, C-11), 65.5 (1C, C-12), 64.2 (1C, C-15), 60.5 (1C, C-5′), 48.9 (1C, C-5), 48.1 (1C, C-13), 43.4 (1C, C-6), 34.4 (1C, C-3), 27.6 (1C, C-8), 25.3 (1C, C-4′), 23.4 (1C, C-16), 20.3 (1C, C-7), 15.7 (1C, C-14′), 7.6 (1C, C-14).
**STLAC (CD$_3$CN)**	
δ [ppm] = 6.92 (bs, 1H, H-9′), 6.54 (s, 1H, H-3′), 4.22 (d, J = 18.4 Hz, 1H, H-8′), 4.20 (d, J = 18.4 Hz, 1H, H-8′), 3.27 (bs, 1H, H-3), 3.19 (d, J = 16.9 Hz, 1H, H-11), 2.83 (d, J = 17.00 Hz, 1H, H-11), 2.05 (m, 1H, H-5), 1.90 (m, 1H, H-1), 1.83 (m, 1H, H-8), 1.75 (m, 1H, H-2), 1.53 (m, 2H, H-7), 1.50 (m, 2H, H-6), 1.43 (m, 1H, H-1), 1.02 (m, 1H, H-2), 1.00 (s, 3H, H-15), 0.93 (s, 3H, H-13), 0.85 (s, 3H, H-14), 0.69 (d, J = 6.6 Hz, 3H, H-12).	δ [ppm] = 171.4 (1C, C-7′), 157.6 (1C, C-6′), 154.2 (1C, C-2′), 135.2 (1C, C-5′), 118.7 (1C, C-1′), 116.6 (1C, C-6′), 101.7 (1C, C-3′), 99.6 (1C, C-9), 75.7 (1C, C-3), 43.2 (1C, C-8′), 40.9 (1C, C-10), 40.7 (1C, C-5), 37.9 (1C, C-4), 37.7 (1C, C-8), 32.6 (1C, C-11), 31.9 (1C, C-7), 28.9 (1C, C-13), 25.9 (1C, C-1), 24.8 (1C, C-2), 22.8 (1C, C-14), 21.7 (1C, C-6), 16.4 (1C, C-15), 15.9 (1C, C-12).
**STLAC AC (C_2D_6OS)**	
δ [ppm] = 9.74 (s, 1H, H-2′), 8.34 (s, 1H, H-9′), 6.56 (s, 1H, H-3′), 4.48 (t, J = 2.8 Hz, 1H, H-8′), 4.25 (d, J = 16.8 Hz, 1H, H-8′), 4.06 (d, J = 16.7 Hz, 1H, H-8′), 3.10 (d, J = 16.9 Hz, 1H, H-11), 2.79 (d, J = 16.9 Hz, 1H, H-11), 2.00 (m, 1H, H-5), 1.99 (s, 3H, H-17), 1.86 (m, 1H, H-6), 1.55 (m, 1H, H-1), 1.53 (m, 1H, H-7), 1.50 (m, 1H, H-2), 1.45 (m, 1H, H-6), 1.42 (m, 2H, H-2), 1.80 (m, 1H, H-8), 1.03 (m, 1H, H-1), 0.98 (s, 3H, H-15), 0.89 (s, 3H, H-13), 0.83 (s, 3H, H-14), 0.67 (d, J = 6.4 Hz, 3H, H-12).	δ [ppm] = 170.1 (1C, C-7′), 169.7 (1C, C-16), 155.9 (1C, C-6′), 153.7 (1C, C-2′), 134.4 (1C, C-4′), 116.3 (1C, C-1′), 114.2 (1C, C-5′), 100.8 (1C, C-3′), 97.5 (1C, C-9), 76.9 (1C, C-3), 41.9 (1C, C-10), 41.7 (1C, C-8′), 40.4 (1C, C-5), 36.4 (1C, C-4), 36.2 (1C, C-8), 31.6 (1C, C-11), 30.6 (1C, C-7), 27.7 (1C, C-13), 24.3 (1C, C-1), 21.9 (1C, C-6), 21.6 (1C, C-14), 20.9 (1C, C-17), 20.2 (1C, C-2), 15.5 (1C, C-15), 15.4 (1C, C-12).
**STCHR B (CD$_3$CN)**	
δ [ppm] = 12.72 (s, 1H, OH-5), 10.01 (s, 1H, H-22), 6.66 (d, J = 10.2 Hz, 1H, H-7), 6.40 (s, 1H, H-4), 5.67 (d, J = 10.2 Hz, 1H, H-8), 5.26 (s, 2H, H-23), 5.12 (t, J = 7.2 Hz, 1H, H-12), 5.07 (t, J = 7.0 Hz, 1H, H-16), 2.09 (m, 2H, H-11), 2.04 (s, 3H, H-25), 2.02 (m, 2H, H-15), 1.93 (m, 2H, H-14), 1.72 (m, 2H, H-10), 1.64 (s, 3H, H-19), 1.57 (s, 3H, H-18), 1.54 (s, 3H, H-20), 1.41 (s, 3H, H-21).	δ [ppm] = 194.9 (1C, C-22), 171.2 (1C, C-24), 161.2 (1C, C-1), 161.2 (1C, C-5), 142.0 (1C, C-3), 136.3 (1C, C-13), 132.2 (1C, C-17), 129.1 (1C, C-8), 125.2 (1C, C-16), 124.7 (1C, C-12), 115.9 (1C, C-2), 113.1 (1C, C-7), 111.3 (1C, C-4), 109.6 (1C, C-6), 82.0 (1C, C-9), 63.3 (1C, C-23), 42.1 (1C, C-10), 40.3 (1C, C-14), 27.5 (1C, C-21), 27.3 (1C, C-15), 25.8 (1C, C-19), 23.3 (1C, C-11), 21.1 (1C, C-25), 17.8 (1C, C-18), 16.0 (1C, C-20).
**STCHR A (CD$_3$CN)**	

δ [ppm] = 12.76 (s, 1H, OH-5), 10.05 (s, 1H, H-22), 6.64 (d, J = 10.2 Hz, 1H, H-7), 6.20 (s, 1H, H-4), 5.61 (d, J = 10.2 Hz, 1H, H-8), 5.11 (t, J = 7.2 Hz, 1H, H-12), 5.07 (t, J = 7.0 Hz, 1H, H-16), 2.49 (s, 3H, H-23), 2.07 (m, 2H, H-11), 2.03 (m, 2H, H-15), 1.94 (m, 2H, H-14), 1.69 (m, 2H, H-10), 1.64 (s, 3H, H-19), 1.57 (s, 3H, H-18), 1.54 (s, 3H, H-20), 1.39 (s, 3H, H-21).	δ [ppm] = 195.2 (1C, C22), 161.6 (1C, C-5), 161.1 (1C, C-1), 145.7, (1C, C-3), 136.3 (1C, C-13), 132.2 (1C, C-17), 128.0 (1C, C-8), 125.2 (1C, C-16), 124.8 (1C, C-12), 116.1 (1C, C-7), 114.1 (1C, C-2), 111.6 (1C, C-4), 107.6 (1C, C-6), 81.6 (1C, C-9), 42.1 (1C, C-10), 40.3 (1C, C-14), 27.4 (1C, C-21), 27.3 (1C, C-15), 25.8 (1C, C-19), 23.3 (1C, C-11), 18.4 (1C, C-23), 17.7 (1C, C-18), 16.0 (1C, C-20).
**STAM (CD$_3$OD)**	
δ [ppm] = 6.73 (s, 1H, H-3), 4.62 (d, J = 17.2 Hz, 1H, H-8′), 4.49 (d, J = 17.2 Hz, 1H, H-8′), 3.85 (m, 2H, H-10′), 3.75 (m, 2H, H-9′), 3.40 (t, 2.7 Hz, 1H, H-3), 3.28 (d, J = 16.9 Hz, 1H, H-11), 2.91 (d, J = 16.9 Hz, 1H, H-11), 2.11 (m, 1H, H-5), 1.88 (m, 2H, H-2, H-1), 1.85 (m, 1H, H-8), 1.60 (m, 5H, H-6, H-1, H-7), 1.14 (m, 1H, H-2), 1.11 (s, 3H, H-15), 1.04 (s, 3H, H-13), 0.94 (s, 3H, H-14), 0.79 (d, J = 6.5 Hz, 3H, H-12).	δ [ppm] = 171.4 (1C, C-7′), 157.5 (1C, C-6′), 155.2 (1C, C-2′), 135.0 (1C, C-5′), 118.7 (1C, C-1′), 114.5 (1C, C-4′), 102.0 (1C-C-3′), 99.7 (1C-C-9), 76.4 (1C, C-3), 61.1 (1C, C-10′), 49.7 (1C, C-8′), 46.3 (1C, C-9′), 43.5 (1C, C-10), 41.4 (1C, C-5), 38.6 (1C, C-4), 28.4 (1C, C-8), 33.0 (1C, C-11), 32.2 (1C, C-7), 29.0 (1C, C-13), 26.0 (1C, C-1), 25.3 (1C, C-2), 23.0 (1C, C-14), 22.1 (1C, C-6), 16.5 (1C, C-15), 15.9 (1C, C-12).
**ST C (CD$_3$CN)**	
δ [ppm] = 10.29 (s, 1H, H-8′), 6.46 (s, 1H, H-3′), 4.77 (d, J = 2.2 Hz, 1H, H-3), 4.64 (s, 2H, H-7′), 3.98 (m, 1H, H-2), 3.14 (d, J = 16.5 Hz, 1H, H-11), 2.83 (d, J = 16.5 Hz, 1H, H-11), 2.09 (m, 1H, H-5), 1.96 (s, 3H, H-17), 1.90 (m, 1H, H-8), 1.64 (m, 1H, H-1), 1.61 (m, 1H, H-7), 1.57 (m, 1H, H-6), 1.50 (m 1H, H-1), 1.48 (m, 1H, H-6), 1.34 (m, 1H, H-7), 1.06 (s, 3H, H-15), 0.96 (s, 3H, H-13), 0.88 (s, 3H, H-14), 0.77 (d, J = 6.5 Hz, 3H, H-12).	δ [ppm] = 189.4 (1C, C-8′), 171.6 (1C, C-16), 169.6 (1C, C-6′), 159.8 (1C C-2′), 146.5 (1C, C-4′), 113.1 (1C, C-1′), 110.8 (1C, C-5\`), 109.1 (1C, C-3′), 100.4 (1C, C-9), 81.0 (1C, C-3), 65.4 (1C, C-2), 64.1 (1C, C-7′), 44.5 (1C, C-10), 41.3 (1C, C-5), 38.9 (1C, C-4), 37.1 (1C, C-8), 34.7 (1C, C-7), 31.9 (1C, C-13) 31.2 (1C, C-1), 28.4 (1C, C-11), 21.9 (1C, C-14), 21.2 (2C, C-6, C-17), 17.2 (1C, C-15), 15.8 (1C, C-12).
**L-671 (CD$_3$CN)**	
δ [ppm] = 10.21 (s, 1H, H-8′), 6.44 (s, 1H, H-3′), 4.63 (d, J = 4.1 Hz, 2H, H-7′), 3.27 (s, 1H, H-3), 3.11 (d, J = 16.4 Hz, 1H, H-11), 2.78 (d, J = 16.4 Hz, 1H, H-11), 2.12 (m, 1H, H-5), 1.92 (m, 1H, H-1), 1.88 (m, 1H, H-8), 1.73 (m, 1H, H-2), 1.60 (m, 1H, H-7), 1.55 (m, 1H, H-6), 1.52 (m, 1H, H-7), 1.48 (m, 1H, H-6), 1.44 (m, 1H, H-1), 1.05 (m, 1H, H-2), 1.01 (s, 3H, H-15), 0.94 (s, 3H, H-13), 0.85 (s, 3H, H-14), 0.73 (d, J = 6.5 Hz, 3H, H-12).	δ [ppm] = 189.5 (1C, C-8′), 169.4 (1C, C-6′), 159.6 (1C, C-2′), 146.3 (1C, C-4′), 113.2 (1C, C-1′), 110.7 (1C, C-5′), 108.9 (1C, C-3′), 101.0 (1C, C-9), 75.6 (1C, C-3), 64.1 (1C, C-7′), 43.3 (1C, C-10), 41.0 (1C, C-5), 38.3 (1C, C-4), 37.8 (1C, C-8), 32.0 (1C, C-7), 31.6 (1C, C-11), 28.9 (1C, C-13), 25.9 (1C, C-1), 24.9 (1C, C-2), 22.7 (1C, C-14), 21.7 (1C, C-6), 16.4 (1C, C-15), 15.9 (1C, C-12).
**STBON D (CD$_3$OD)**	
δ [ppm] = 10.25 (s, 1H, H-8′), 6.59 (s, 1H, H-3′), 5.23 (m, 1H, H-2), 4.93 (d, J = 2.2 Hz, 1H, H-3), 4.81 (d, J = 6.1 Hz, 2H, H-7′), 3.13 (d, J = 16.5 Hz, 1H, H-11), 2.86 (d, J = 16.5 Hz, 1H, H-11), 2.17 (m, 1H, H-5), 2.00 (s, 3H, H-19), 1.93 (m, 1H, H-8), 1.87 (s, 3H, H-17), 1.85 (m, 1H, H-1), 1.70 (m, 1H, H-7), 1.61 (m, 1H, H-6), 1.54 (m, 2H, H-6, H-7), 1.40 (m, 1H, H-1), 1.16 (s, 3H, H-15), 1.05 (s, 3H, H-13), 0.94 (s, 3H, H-14), 0.81 (d, J = 6.5 Hz, 3H, H-12).	δ [ppm] = 189.3 (1C; C-7′), 172.4 (1C, C-18), 172.3 (1C, C-16); 169.8 (1C, C-6′), 161.6 (1C, C-2′), 147.2 (1C, C-4′), 112.8 (1C, C-1′), 110.2 (1C, C-5′), 109.0 (1C, C-3′), 100.4 (1C, C-9), 78.4 (1C, C-3), 69.6 (1C, C-2), 64.0 (1C, C-8′), 45.0 (1C, C-10), 42.1 (1C, C-5), 39.2 (1C, C-4), 37.7 (1C, C-8), 32.3 (1C, C-7), 31.6 (2C, C-1,C-11), 28.4 (1C, C-13), 21.9 (1C, C-14), 21.5 (1C, C-6), 21.0 (1C, C-17), 20.8 (1C, C-19), 17.3 (1C, C-15), 15.8 (1C, C-12).
**STDIAL (CD$_3$CN)**	
δ [ppm] = 10.50 (s, 1H, H-8′), 10.33 (s, 1H, H-7′), 6.77 (s, 1H, H-3′), 3.28 (t, J = 2.5 Hz, 1H, H-3), 3.18 (d, J = 16.9 Hz, 1H, H-11), 2.85 (d, J = 17.5 Hz, 1H, H-11), 2.11 (m, 1H, H-5), 1.92 (m, 1H, H-1), 1.91 (m, 1H, H-8), 1.74 (m, 1H, H-2), 1.62 (m, 1H, H-1), 1.56 (m, 1H, H-6), 1.53 (m, 1H, H-7), 1.48 (m, 1H, H-6), 1.47 (m, 1H, H-1), 1.07 (m, 1H, H-2), 1.02 (s, 3H, H-15), 0.94 (s, 3H, H-13), 0.85 (s, 3H, H-14), 0.74 (d, J = 6.6 Hz, 3H, H-12).	δ [ppm] = 193.4 (1C, C-8′), 189.0 (1C, C-7′), 168.2 (1C, C-6′), 158.7 (1C, C-2′), 139.6 (1C, C-4′), 120.2 (1C, C-1′), 112.3 (1C, C-5′), 109.2 (1C, C-3′), 101.7 (1C, C-9), 75.6 (1C, C-3), 43.3 (1C, C-10), 41.0 (1C, C-5), 38.3 (1C, C-4), 37.8 (1C, C-8), 31.9 (1C, C-7), 31.5 (1C, C-11), 28.9 (1C, C-13), 25.9 (1C, C-1), 24.9 (1C, C-2), 22.7 (1C, C-14), 21.7 (1C, C-6), 16.4 (1C, C-15), 15.9 (1C, C-12).
**ST B (CD$_3$CN)**	
δ [ppm] = 10.31 (s, 1H, H-8′), 6.48 (s, 1H, H-3′), 4.65	δ [ppm] = 189.3 (1C, C-8′), 171.0 (1C, C-16), 169.7 (1C,

(bs, 2H, H-7′), 4.55 (bs, 1H, H-3), 3.13 (d, J = 16.5 Hz, 1H, H-11), 2.83 (d, J = 16.5 Hz, 1H, H-11), 2.20 (m, 1H, H-5), 1.93 (s, 3H, H-17), 1.92 (m, 1H, H-8), 1.91 (m, 1H, H-2), 1.65 (m, 1H, H-1), 1.64 (m, 1H, H-7), 1.59 (m, 2H, H-6), 1.55 (, 1H, H-2), 1.53 (m, 1H, H-7), 1.15 (m, 1H, H-1), 1.05 (s, 3H, H-15), 0.95 (s, 3H, H-13), 0.90 (s, 3H, H-14), 0.78 (d, J = 6.4 Hz, 3H, H-12).	C-6′), 159.5 (1C, C-2′), 146.5 (1C, C-4′), 113.2 (1C, C-1′), 110.8 (1C, C-5′), 108.8 (1C, C-3′), 99.8 (1C, C-9), 78.4 (1C, C-3), 64.0 (1C, C-7′), 43.2 (1C, C-10), 42.0 (1C, C-5), 38.0 (1C, C-4), 37.5 (1C, C-8), 31.9 (1C, C-7), 31.1 (1C, C-11), 28.3 (1C, C-13), 25.6 (1C, C-1), 23.0 (1C, C-2), 22.1 (1C, C-14), 21.5 (1C, C-6), 21.1 (1C, C-17), 16.3 (1C, C-15), 15.8 (1C, C-12).
**ACDIAL AC (CD_3CN)**	
δ [ppm] = 10.49 (s, 1H, H-8′), 10.41 (s, 1H, H-7′), 6.82 (s, 1H, H-3′), 5.19 (m, 1H, H-2), 4.89 (d, J = 1.8 Hz, 1H, H-3), 3.21 (d, J = 17.5 Hz, 1H, H-11), 2.93 (d, J = 17.5 Hz, 1H, H-11), 2.05 (m, 1H, H-5), 2.02 (s, 3H, H-19), 1.91 (m, 1H, H-8), 1.81 (s, 3H, H-17), 1.79 (m, 1H, H-1), 1.65 (m, 1H, H-7), 1.57 (m, 1H, H-6), 1.49 (m, 2H, H-6, H-7), 1.39 (m, 1H, H-1), 1.11 (s, 3H, H-15), 1.02 (s, 3H, H-13), 0.90 (s, H, H-14), 0.75 (d, J = 6.5 Hz, 3H, H-12).	δ [ppm] = 193.4 (1C, C-8′), 188.9 (1C, C-7′), 171.5 (1C, C-18), 171.1 (1C, C-16), 167.6 (1C, C-6′), 156.4 (1C, C-2′), 128.5 (1C, C-4′), 120.7 (1C, C-1′), 112.6 (1C, C-5′), 109.7 (1C, C-3′), 100.9 (1C, C-9), 77.4 (1C, C-3), 68.9 (1C, C-2), 44.6 (1C, C-10), 41.5 (1C, C-5), 38.8 (1C, C-4), 37.1 (1C, C-8), 32.6 (1C, C-7), 31.5 (1C, C-11), 31.2 (1C, C-1), 28.2 (1C, C-14), 21.7 (1C, C-13), 21.1 (3C, C-17, C-19), 17.1 (1C, C-15), 15.7 (1C, C-12).
**STDIAL AC (CD_3CN)**	
δ [ppm] = 10.49 (s, 1H, H-8′), 10.39 (s, 1H, H-7′), 6.77 (s, 1H, H-3′), 4.53 (t, J = 2.9 Hz, 1H, H-3), 3.17 (d, J = 17.5 Hz, 1H, H-11), 2.88 (d, J = 17.5 Hz, 1H, H-11), 2.19 (m, 1H, H-5), 1.97 (d, J = 6.5 Hz, 1H, H-8), 1.92 (s, 3H, H-17), 1.89 (m, 1H, H-2), 1.64 (m, 1H, H-7), 1.63 (m, 1H, H-1), 1.58 (m, 1H, H-6), 1.53 (m, 1H, H-2), 1.52 (m, 2H, H-6, H-7), 1.13 (m, 1H, H-1), 1.04 (s, 3H, H-15), 0.94 (s, 3H, H-13), 0.90 (s, 3H, H-14), 0.77 (s, 3H, H-12).	δ [ppm] = 193.4 (1C, C-8′), 188.9 (1C, C-7′), 171.1 (1C, C-16), 168.0 (1C, 6′), 158.7 (1C, C-2′), 139.7 (1C, 4′), 120.1 (1C, C-1′), 112.4 (1C, C-5′), 109.3.0 (1C, C-3′), 101.5 (1C, C-9), 78.4 (1C, C-3), 43.3 (1C, C-10), 41.9 (1C, C-5), 37.5 (2C, C-4, C-8), 31.9 (1C, C-7), 31.4 (1C, C-11), 28.3 (1C, C-14), 25.5 (1C, C-1), 23.0 (1C, C-2), 22.0 (1C, 13), 21.5 (1C, C-6), 21.2 (1C, C-17), 16.3 (1C, C-15), 15.8 (1C, C-12).
**STCHR C (CD_3CN)**	
δ [ppm] = 12.95 (s, 1H, OH-5), 10.63 (s, 1H, H-22), 10.02 (s, 1H, H-23), 6.90 (s, 1H, H-4), 6.71 (d, J = 10.2 Hz, 1H, H-7), 5.82 (d, J = 10.2 Hz, 1H, H-8), 5.12 (t, J = 7.2 Hz, 1H, H-12), 5.07 (t, J = 7.0 Hz, 1H, H-16), 2.12 (m, 2H, H-11), 2.03 (m, 2H, H-15), 1.94 (m, 2H, H-14), 1.77 (m, 2H, H-10), 1.64 (s, 3H, H-19), 1.57 (s, 3H, H-18), 1.54 (s, 3H, H-20), 1.45 (s, 3H, H-21).	δ [ppm] = 196.6 (1C, C-22), 193.5 (1C, C-23), 161.5 (1C, C-1), 160.4 (1C, C-5), 138.8 (1C, C-3), 136.5 (1C, C-13), 132.2 (1C, C-17), 131.8 (1C, C-8), 125.2 (1C, C-16), 124.6 (1C, C-12), 117.5 (1C, C-4), 115.8 (1C, C-7), 114.0 (1C, C-6), 113.1 (1C, C-2), 82.4 (1C, C-9), 42.1 (1C, C-10), 40.3 (1C, C-14), 27.5 (1C, C-21), 27.3 (1C, C-15), 25.8 (1C, C-19), 23.2 (1C, C-11), 17.7 (1C, C-18), 16.0 (1C, C-20).

2. LC-MS/MS Data

Table S3. SRM parameters for all 15 analytes (tR retention time, DP declustering potential, CE collision energy.

Analyte	Parent ion [m/z]	t_R [min]	Quantifier/qualifier [Da]	DP [V]	CE [V]
Sat G	$[M + Na]^+$ 567	1.95	263/231	200	35/35
Sat H	$[M + Na]^+$ 551	2.15	303/321	200	35/35
Stlac	$[M + H]^+$ 386	3.10	178/150	80	50/60
Stlac ac	$[M + H]^+$ 428	3.70	178/216	230	42/41
Stchr B	$[M + H]^+$ 413	5.50	353/163	60	20/40
Stchr A	$[M + H]^+$ 355	5.95	165/205	80	30/20
Stam	$[M-H]^-$ 428	2.20	221/191	−100	-50/-60
St C	$[M-H]^-$ 445	2.65	385/179	−240	−45/−60
L-671	$[M-H]^-$ 387	3.10	151/179	−220	−55/−45
Stbon D	$[M-H]^-$ 487	3.50	179/151	−220	−70/−70
Stdial (lactone)	$[M-H]^-$ 385	3.66	313/341	−220	−40/−45
Stdial (dial)	$[M-H]^-$ 385	3.80	150/122	−220	−50/−55
St B	$[M-H]^-$ 429	4.05	369/151	−220	−51/−70
Acdial ac (lactone)	$[M-H]^-$ 485	4.15	399/357	−220	−40/−45
Acdial ac (dial)	$[M-H]^-$ 485	4.20	365/150	−220	−45/−60
Stdial ac (lactone)	$[M-H]^-$ 427	4.65	383/323	−210	−45/−55
Stdial ac (dial)	$[M-H]^-$ 427	4.75	367/150	−210	−45/−55
Stchr C	$[M-H]^-$ 367	6.55	150/161	−150	−40/−45

Table S4. LODs, LOQs, and working ranges in ng/mL of the 15 *Stachybotrys* analytes.

Analyte	LOD [ng/mL]	LOQ [ng/mL]	Working range [ng/mL]
SAT G	3.2	14	14–1414
SAT H	2.4	11	11–1061
STLAC	0.60	2.7	3–298
STLAC AC	0.040	0.18	0.2–19.8
STCHR B	0.24	1.1	1–99
STCHR A	0.20	0.90	1–99
STAM	0.30	1.3	1.5–148.5
ST C	0.15	0.68	1–99
L-671	0.25	1.1	1–99
STBON D	0.15	0.67	1–99
STDIAL	0.086	0.39	0.5–49.5
ST B	0.24	1.1	1–99
ACDIAL AC	0.082	0.37	0.5–49.5
STDIAL AC	0.023	0.11	0.1–9.9
STCHR C	1.5	5.0	5–502

3. Growth Progression and Macromorphology

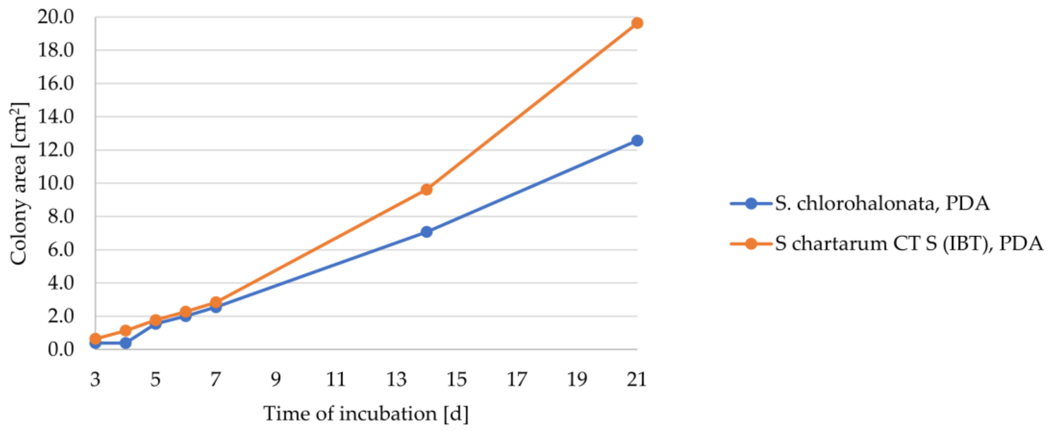

Figure S1. Colony areas in cm^2 of *S. chlorohalonata* CBS 109283 and *S. chartarum* CT S IBT 40293 grown on PDA (time 3, 4, 5, 6, 7, 14, and 21 days).

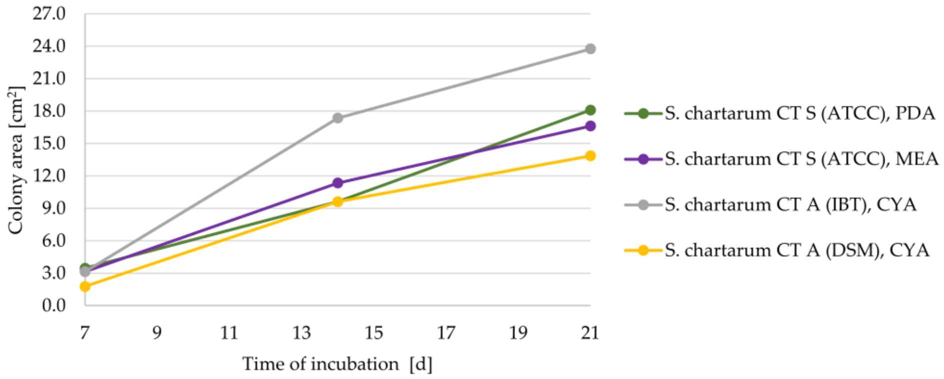

Figure S2. Colony areas in cm² of *S. chartarum* (CT S) IBT 40293 grown on PDA, *S. chartarum* (CT S) ATCC 34916 grown on MEA and *S. chartarum* (CT A) IBT 40288 and *S. chartarum* (CT A) DSM 63425 grown on CYA (time 7, 14, and 21 days).

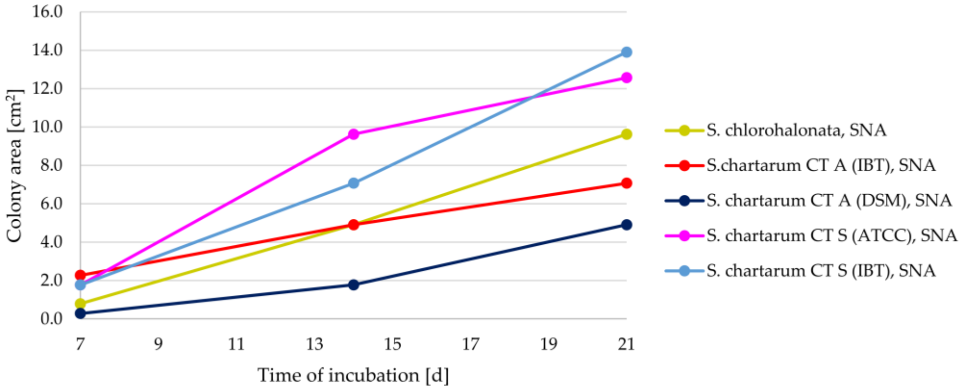

Figure S3. Colony areas in cm² of *S. chlorohalonata* CBS 109283, *S. chartarum* CT A IBT 40288, *S. chartarum* CT A DSM 63425, *S. chartarum* CT S ATCC 34916 grown on MEA and *S. chartarum* CT S IBT 40293 grown on SNA (time 7, 14, and 21 days).

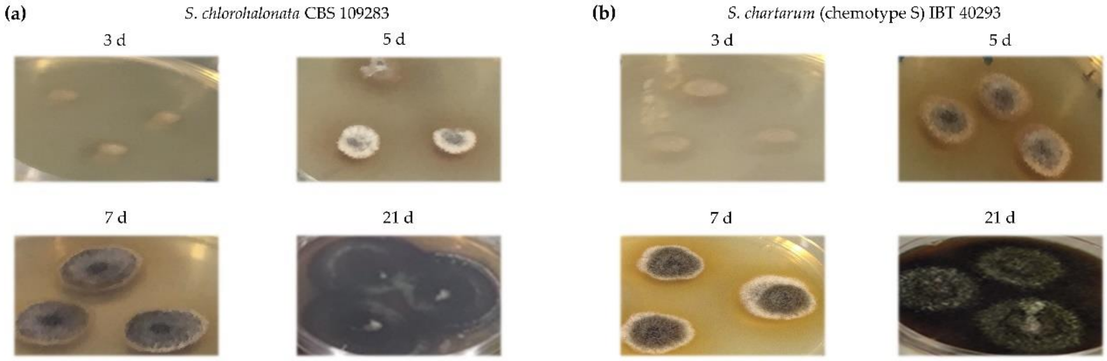

Figure S4. Macromorphology of *S. chlorohalonata* CBS 109283 **(a)** and *S. chartarum* (CT S) IBT 40293 **(b)** on PDA after 3, 5, 7, and 21 days of cultivation at 25 °C in the dark.

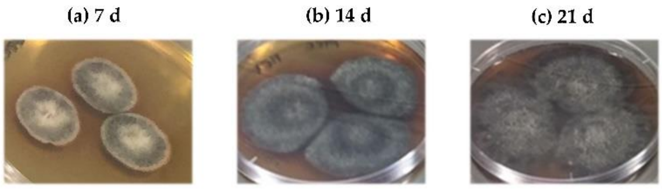

Figure S5. Macromorphology of *S. chartarum* (CT S) ATCC 34916 on MEA after 7 days **(a)**, 14 days **(b)** and 21 days **(c)** of cultivation at 25 °C in the dark.

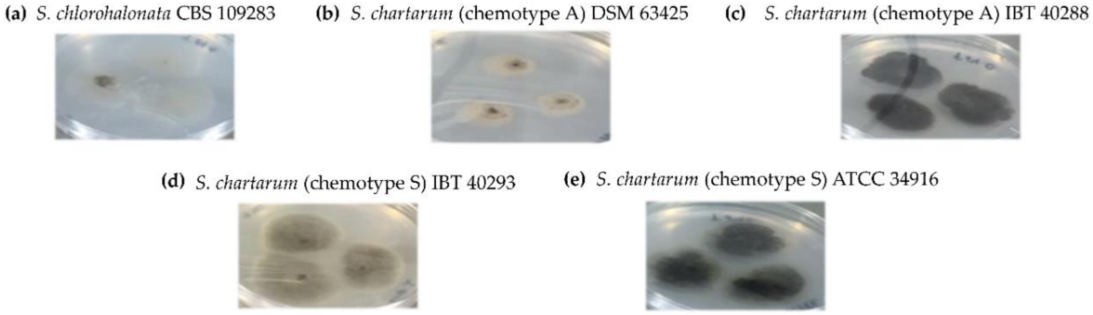

Figure S6. Macromorphology of *S. chlorohalonata* CBS 109283 **(a)**, *S. chartarum* (CT A) DSM 63425 **(b)**, *S. chartarum* (CT A) IBT 40288 **(c)**, *S. chartarum* (CT S) IBT 40293 **(d)** and S. *chartarum* (CT S) ATCC 34916 **(e)** on SNA after 14 days of cultivation at 25 °C in the dark.

4. Data Visualization and Interpretation

The following aspects were considered for data interpretation:

Relative (pie charts) and absolute levels (bar charts) of determined metabolites are described and interpreted. The relative profile represents the percentage of the individual metabolite relative to the total amount of metabolites (100%).

It should be noted that this study deals only with 15 (major) secondary metabolites and the formation of further metabolites is expected. These 15 analytes can be either intermediates or final products within the biosynthesis. Therefore, it can lead to a decrease of the respective metabolite level within this targeted method. In some cases, metabolite concentrations/portions are presented as the $\sum$ < LOD/LOQ, due to lower levels and dilution steps.

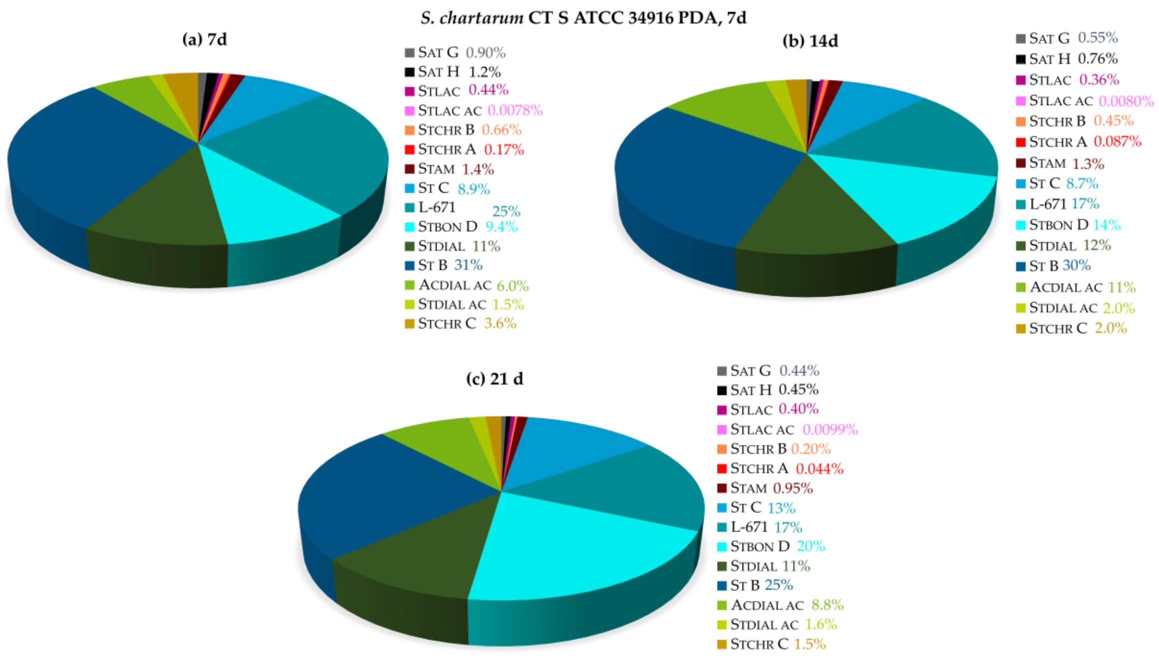

Figure S7. Relative secondary metabolite profiles of *S. chartarum* CT S ATCC 34916 on PDA after 7 days **(a)**, 14 days **(b)** and 21 days **(c)** of cultivation at 25 °C in the dark.

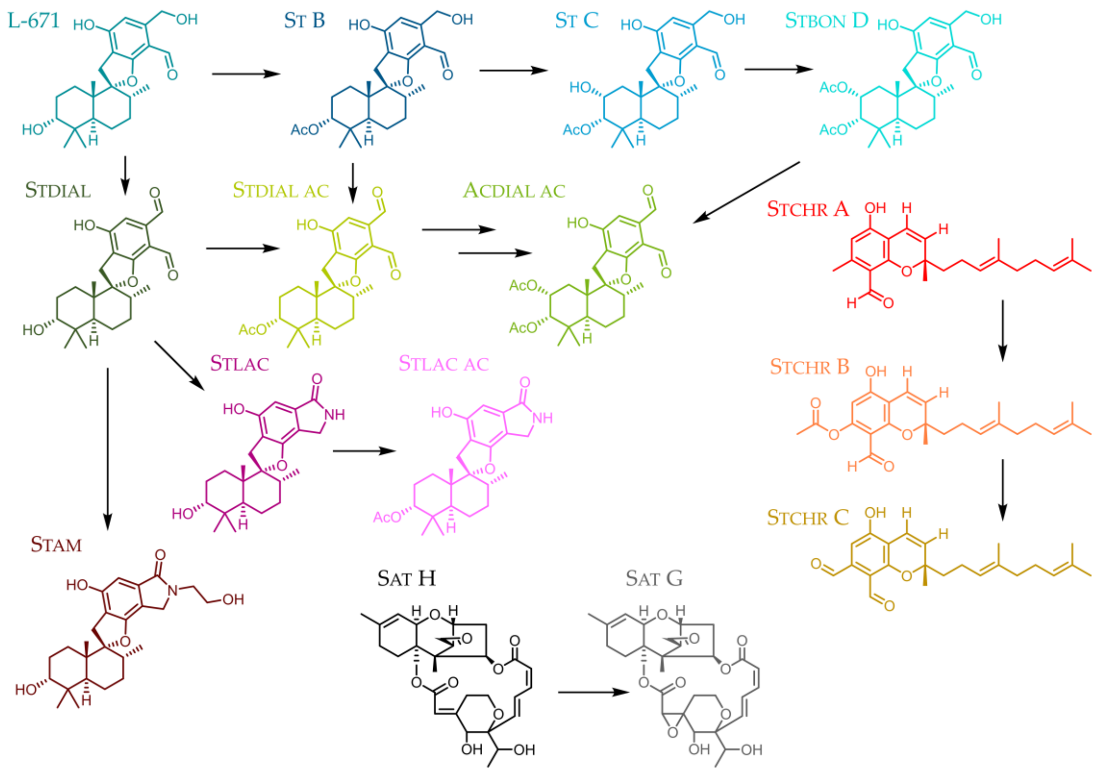

Figure S8. Proposed biosynthetic pathways of secondary metabolites produced by *Stachybotrys* spp.
